# Non-noble metal-nitride based electrocatalysts for high-performance alkaline seawater electrolysis

**DOI:** 10.1038/s41467-019-13092-7

**Published:** 2019-11-08

**Authors:** Luo Yu, Qing Zhu, Shaowei Song, Brian McElhenny, Dezhi Wang, Chunzheng Wu, Zhaojun Qin, Jiming Bao, Ying Yu, Shuo Chen, Zhifeng Ren

**Affiliations:** 1 College of Physical Science and Technology, Central China Normal University, Wuhan, 430079 China; 2 Department of Physics and TcSUH, University of Houston, Houston, TX 77204 USA; 3 Materials Science and Engineering Program, University of Houston, Houston, TX 77204 USA; 4 Department of Electrical and Computer Engineering, University of Houston, Houston, TX 77204 USA

**Keywords:** Electrocatalysis, Materials chemistry, Electrocatalysis, Nanoscale materials

## Abstract

Seawater is one of the most abundant natural resources on our planet. Electrolysis of seawater is not only a promising approach to produce clean hydrogen energy, but also of great significance to seawater desalination. The implementation of seawater electrolysis requires robust and efficient electrocatalysts that can sustain seawater splitting without chloride corrosion, especially for the anode. Here we report a three-dimensional core-shell metal-nitride catalyst consisting of NiFeN nanoparticles uniformly decorated on NiMoN nanorods supported on Ni foam, which serves as an eminently active and durable oxygen evolution reaction catalyst for alkaline seawater electrolysis. Combined with an efficient hydrogen evolution reaction catalyst of NiMoN nanorods, we have achieved the industrially required current densities of 500 and 1000 mA cm^−2^ at record low voltages of 1.608 and 1.709 V, respectively, for overall alkaline seawater splitting at 60 °C. This discovery significantly advances the development of seawater electrolysis for large-scale hydrogen production.

## Abstract (web summary)

Seawater electrolysis is a promising approach to produce hydrogen fuel and is also of great significance to seawater desalination. Here, the authors prepare 3D core-shell metal-nitride catalysts from earth-abundant elements for high-performance alkaline seawater electrolysis.

## Introduction

Hydrogen (H_2_) is playing an increasingly important role as an ideal energy source owing to its high energy density (142 MJ kg^−1^) and pollution-free use^1–5^. Splitting water into H_2_ and oxygen (O_2_) by electricity produced from waste heat or from renewable but intermittent wind or solar energy is one of the most efficient and sustainable routes for high-purity H_2_ production^6–11^. Over the past decade, many low-cost water electrolyzers with electrolytes consisting of high-purity freshwater have been developed, and some achieve performance even better than that of the benchmark platinum (Pt) and iridium dioxide (IrO_2_) catalysts^12–15^. However, large-scale freshwater electrolysis would put a heavy strain on vital water resources. Seawater is one of the most abundant natural resources on our planet and accounts for 96.5% of the world’s total water resources^16^. Direct electrolysis of seawater rather than freshwater is highly significant, especially for the arid zones, since this technology not only stores clean energy, but also produces fresh drinking water from seawater. Nevertheless, the implementation of seawater splitting remains highly challenging, especially for the anodic reaction.

The major challenge in seawater splitting is the chlorine evolution reaction (CER), which occurs on the anode due to the existence of chloride anions (∼0.5 M) in seawater, and competes with the oxygen evolution reaction (OER)^17,18^. For the CER in alkaline media, chlorine would further react with OH^−^ for hypochlorite formation with an onset potential of about 490 mV higher than that of OER, and thus highly active OER catalysts are demanded to deliver large current densities (500 and 1000 mA cm^−2^) at overpotentials well below 490 mV to avoid hypochlorite formation^18,19^. Another bottleneck hindering the progress of seawater splitting is the formation of insoluble precipitates, such as magnesium hydroxide, on the electrode surface, which may poison the OER and hydrogen evolution reaction (HER) catalysts^19^. To alleviate this issue, catalysts possessing large surface areas with numerous active sites are more favorable. In addition, the aggressive chloride anions in seawater also corrode the electrodes, further restricting the development of seawater splitting^18^. Because of these intractable obstacles, only a few studies on electrocatalysts for seawater splitting have been reported, with limited progress made thus far. Recently, Kuang et al. reported an impressive anode catalyst composed of a nickel-iron hydroxide layer coated on a nickel sulfide layer for active and stable alkaline seawater electrolysis, in which a current density of 400 mA cm^−2^ was achieved at 1.72 V for two-electrode electrolysis in 6 M KOH + 1.5 M NaCl electrolyte at 80 °C^18^. Other non-precious electrocatalysts, including transition metal hexacyanometallate, cobalt selenide, cobalt borate, and cobalt phosphate, have been well studied for OER in NaCl-containing electrolytes^17,20,21^, but the overpotentials needed to deliver large current densities (500 and 1000 mA cm^−2^) are much higher than 490 mV, not to mention the activity for overall seawater splitting. Therefore, it is highly desirable to develop other robust and inexpensive electrocatalysts to expedite the sluggish seawater splitting process, especially for OER at large current densities, so as to boost research on large-scale seawater electrolysis.

Transition metal-nitride (TMN) is highly corrosion-resistant, electrically conductive, and mechanically strong, which makes it a very promising candidate for electrolytic seawater splitting^22^. Recent studies on Ni_3_N/Ni, NiMoN, and Ni-Fe-Mo trimetallic nitride catalysts have established TMN-based materials to be efficient non-noble metal electrocatalysts for freshwater splitting in alkaline media (1 M KOH)^23–25^. Considering the need for catalysts with large surface areas and high-density active sites for seawater splitting, here we report the design and synthesis of a three-dimensional (3D) core-shell TMN-based OER electrocatalyst, in which NiFeN nanoparticles are uniformly decorated on NiMoN nanorods supported on porous Ni foam (NiMoN@NiFeN) for exceptional alkaline seawater electrolysis. The 3D core-shell catalyst yields large current densities of 500 and 1000 mA cm^−2^ at overpotentials of 369 and 398 mV, respectively, for OER in 1 M KOH + natural seawater at 25 °C. In-depth studies show that in situ evolved amorphous layers of NiFe oxide and NiFe oxy(hydroxide) on the anode surface are the real active sites that are not only responsible for the excellent OER performance, but also contribute to the superior chlorine corrosion-resistance. Additionally, the integrated 3D core-shell TMN nanostructures with multiple levels of porosity offer numerous active sites, efficient charge transfer, and rapid gaseous product releasing, which also account for the promoted OER performance. An outstanding two-electrode seawater electrolyzer has subsequently been fabricated by pairing this OER catalyst with another efficient HER catalyst of NiMoN, where the current densities of 500 and 1000 mA cm^−2^ are achieved at record low voltages of 1.608 and 1.709 V, respectively, for overall alkaline seawater splitting at 60 °C, along with superior stability. Impressively, our electrolyzer can be driven by an AA battery or a commercial thermoelectric (TE) module, demonstrating great potential and flexibility in utilizing a broad range of power sources. Overall, this work greatly boosts the science and technology of seawater electrolysis.

## Results

### Electrocatalyst preparation and characterization

**Fig. 1:**
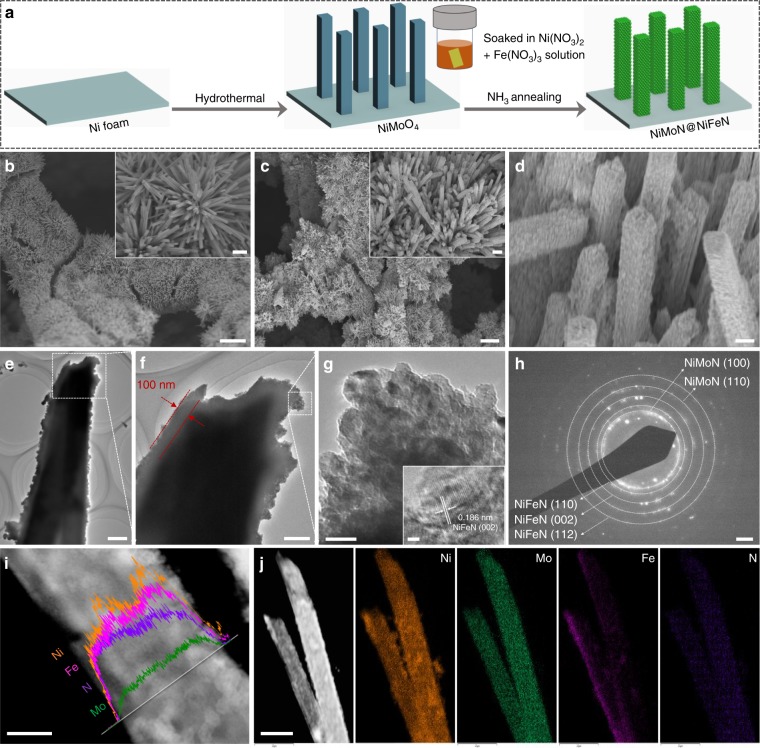
Synthesis and microscopic characterization of the as-prepared NiMoN@NiFeN catalyst. **a** Schematic illustration of the synthesis procedures for the self-supported 3D core-shell NiMoN@NiFeN catalyst. **b**–**d** SEM images of (**b**) NiMoN and (**c**, **d**) NiMoN@NiFeN at different magnifications. **e**, **f** TEM images of NiMoN@NiFeN core-shell nanorods at different magnifications. **g** HRTEM image, **h** SAED pattern, **i** EDS line scan, and **j** dark field scanning transmission electron microscopy (DF-STEM) image and corresponding elemental mapping of the NiMoN@NiFeN catalyst. Scale bars: **b**, **c** 30 µm; insets of (**b**, **c**) 3 µm; **d**, **e** 500 nm; **f** 200 nm; **g** 20 nm; inset of (**g**) 1 nm; **h** 2 1/nm; **i** 250 nm; **j** 1 µm

Figure 1a presents a schematic illustration of the synthesis procedures for the 3D core-shell NiMoN@NiFeN catalyst, where commercial Ni foam (Supplementary Fig. 1) is used as the conductive support due to its high surface area, good electrical conductivity, and low cost^26^. We first used a hydrothermal method to synthesize NiMoO_4_ nanorod arrays on Ni foam, which was then soaked in a NiFe precursor ink and air-dried, followed by a one-step thermal nitridation. The stable construction and the hydrophilic nature of the NiMoO_4_ nanorod arrays (Supplementary Fig. 2) facilitate the uniform coverage of the nanorods by the NiFe precursor ink. The pure NiMoN catalyst was prepared by nitridation of NiMoO_4_ without soaking in the precursor ink, and scanning electron microscopy (SEM) images show that numerous nanorods with smooth surfaces were uniformly and vertically grown on the surface of the Ni foam (Fig. 1b and its inset, and Supplementary Fig. 3). After soaking in the precursor ink and thermal nitridation, the NiMoN@NiFeN shows a well-preserved nanorod morphology with rough and dense surfaces (Fig. 1c and its inset). The high-magnification SEM image in Fig. 1d clearly shows that the surfaces of the nanorods were uniformly decorated with many nanoparticles, forming a unique 3D core-shell nanostructure that offers an extremely large surface area with a huge quantity of active sites, even with the formation of insoluble precipitates during seawater electrolysis. For comparison, pure NiFeN nanoparticles (Supplementary Fig. 4) were also synthesized on the Ni foam by soaking bare Ni foam in the NiFe precursor ink, followed by thermal nitridation. We also studied the morphology variation of NiMoN@NiFeN with different loading amounts of NiFeN nanoparticles by controlling the concentration of NiFe precursor ink (Supplementary Fig. 5). It was determined that the optimized concentration is 0.25 g ml^−1^, so this concentration was used for further analyses unless otherwise indicated.

Transmission electron microscopy (TEM) images of NiMoN@NiFeN in Fig. 1e, f further detail the desired core-shell morphology of the nanoparticle-decorated nanorods, showing that the thickness of the NiFeN shell is about 100 nm. Figure 1g displays a high-resolution TEM (HRTEM) image taken from the tip of the NiMoN@NiFeN nanorod presented in Fig. 1f, showing that the NiFeN nanoparticles are highly mesoporous and interconnected with one another to form a 3D porous network, which is beneficial for seawater diffusion and gaseous product release^27,28^. The HRTEM image in the Fig. 1g inset reveals distinctive lattice fringes with interplanar spacings of 0.186 nm, which is assigned to the (002) plane of NiFeN. The selected area electron diffraction (SAED) pattern (Fig. 1h) recorded from the NiMoN@NiFeN core-shell nanorod exhibits apparent diffraction rings of NiMoN and NiFeN, confirming the existence of NiMoN and NiFeN phases. The energy dispersive X-ray spectroscopy (EDS) line scan result (Fig. 1i) and EDS mapping analysis (Fig. 1j) further verify the quintessential core-shell nanostructure, clearly displaying that Mo and Fe are distributed in the central nanorod and edge nanoparticles, respectively, while Ni and N are homogeneously distributed throughout the entire core-shell nanorod.

**Fig. 2:**
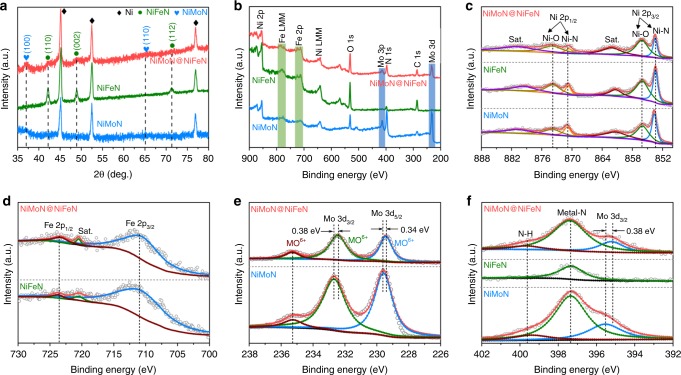
Structural characterization of as-prepared catalysts. **a** XRD, and **b** XPS survey, and **c**–**f** high-resolution XPS of (**c**) Ni 2p, (**d**) Fe 2p, (**e**) Mo 3d, and (**f**) N 1 s of the NiMoN, NiFeN, and NiMoN@NiFeN catalysts

We then conducted X-ray diffraction (XRD) and X-ray photoelectron spectroscopy (XPS) measurements to study the chemical compositions and surface element states of the catalysts. Typical XRD patterns (Fig. 2a) reveal the successful formation of NiMoN and NiFeN compositions after corresponding thermal nitridation. Figure 2b shows the XPS survey spectra, demonstrating the presence of Ni, Mo, and N in the NiMoN nanorods; Ni, Fe, and N in the NiFeN nanoparticles; and Ni, Mo, Fe, and N in the core-shell NiMoN@NiFeN nanorods. For the high-resolution XPS of Ni 2p of the three catalysts (Fig. 2c), the two peaks at 853.4 and 870.8 eV are attributed to the Ni 2p_3/2_ and Ni 2p_1/2_ of Ni species in Ni-N, respectively, while the peaks located at 856.3 and 873.9 eV are assigned to the Ni 2p_3/2_ and Ni 2p_1/2_ of the oxidized Ni species (Ni–O), respectively^29^. The two additional peaks at 862.0 and 880.1 eV are the relevant satellite peaks (Sat.). The Fe 2p XPS of NiFeN and NiMoN@NiFeN in Fig. 2d show two peaks of Fe 2p_3/2_ and Fe 2p_1/2_ at 711.0 and 723.6 eV, respectively, as well as a tiny peak at 720.5 corresponding to the satellite peak^30^. In Fig. 2e, the Mo 3d XPS of NiMoN and NiMoN@NiFeN show two valence states of Mo^3+^ and Mo^6+^. For NiMoN, the peak located at 229.6 eV (Mo 3d_5/2_) is ascribed to Mo^3+^ in the metal-nitride, which is recognized to be active for HER^22^. The peaks at 232.7 (Mo 3d_3/2_) and 235.3 eV are attributed to Mo^6+^ due to the surface oxidation of NiMoN^31^. However, the two main peaks of Mo 3d_5/2_ (Mo^3+^) and Mo 3d_3/2_ (Mo^6+^) show an apparent negative shift in binding energy for the NiMoN@NiFeN, indicating the strong electronic interactions between NiMoN and NiFeN. For the N 1 s XPS (Fig. 2f), the main peak is located at 397.4 eV, which is ascribed to the N species in metal-nitrides, and another peak at 399.6 eV originates from the incomplete reaction of NH_3_^23,32^. Additionally, the Mo 3p_3/2_ peak also appears for the NiMoN and NiMoN@NiFeN, and a negative shift in binding energy still exists for the NiMoN@NiFeN, which is in good agreement with the results in Fig. 2e.

### Oxygen and hydrogen evolution catalysis

**Fig. 3:**
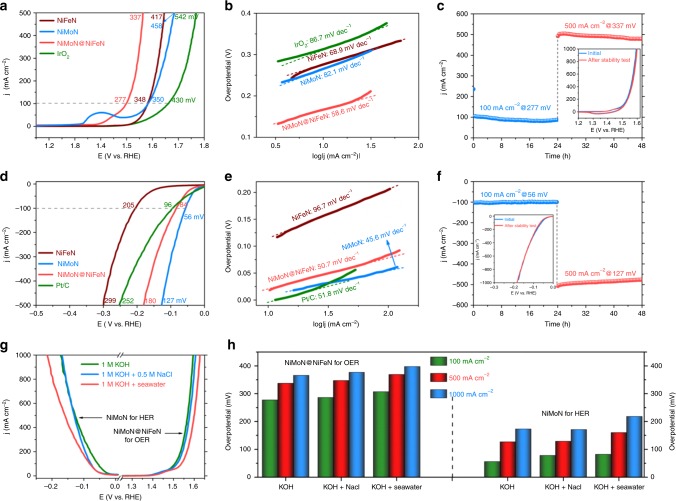
Oxygen and hydrogen evolution catalysis. **a** OER polarization curves in 1 M KOH, and **b** corresponding Tafel plots of different catalysts. **c** OER chronoamperometry curves of NiMoN@NiFeN at overpotentials of 277 and 337 mV in 1 M KOH. Inset: CV curves of NiMoN@NiFeN before and after the stability test. **d** HER polarization curves tested in 1 M KOH, and **e** corresponding Tafel plots of different catalysts. **f** HER chronoamperometry curves of NiMoN at overpotentials of 56 and 127 mV in 1 M KOH. Inset: LSV curves of NiMoN before and after the stability test. **g** OER and HER polarization curves of NiMoN@NiFeN and NiMoN, respectively, in different electrolytes. **h** Comparison of the overpotentials required to achieve current densities of 100, 500, and 1000 mA cm^−2^ for NiMoN@NiFeN (OER) and NiMoN (HER) in different electrolytes

We first evaluated the OER activity of the as-prepared catalysts in 1 M KOH electrolyte in freshwater at room temperature (25 °C). The benchmark IrO_2_ catalyst on Ni foam was also included for comparison. All of the measured potentials *vs*. Hg/HgO were converted to the reversible hydrogen electrode (RHE) according to the reference electrode calibration (Supplementary Fig. 6, *E*_RHE_ = *E*_Hg/HgO _+ 0.925). All data were measured after cyclic voltammetry (CV) activation and reported with iR compensation (85%). The current density was normalized by the geometrical surface area unless otherwise mentioned. As the CV forward scan results in Fig. 3a show, our 3D core-shell NiMoN@NiFeN catalyst exhibits significantly improved OER activity, requiring overpotentials as low as 277 and 337 mV to achieve current densities of 100 and 500 mA cm^−2^, respectively, which are considerably smaller than that of NiFeN (348 and 417 mV), NiMoN (350 and 458 mV), and the benchmark IrO_2_ electrodes (430 and 542 mV). This performance is also superior to that of most non-precious OER catalysts in 1 M KOH (Supplementary Table 1), including the recently reported ZnCo oxyhydroxide^33^, Se-doped FeOOH^34^, NiCoFe-MOF (metal-organic frameworks)^35^, and FeNiP/NCH (nitrogen-doped carbon hollow framework)^36^. The polarization curves of the CV backward scan, the CV without and with iR compensation are presented for comparison in Supplementary Figs. 7, 8, and 9a, respectively. We also investigated the redox behaviors of the different metal-nitride catalysts by analyzing their CV curves in the range of about 1.125 ~1.525 V vs. RHE, and the results are displayed in Supplementary Fig. 9b–d. In addition, the OER activity of other NiMoN@NiFeN catalysts with different loading amounts of NiFeN was also studied (Supplementary Fig. 10), and the one prepared with a precursor ink concentration of 0.25 g ml^−1^ exhibits the highest OER activity. Tafel plots in Fig. 3b show that the NiMoN@NiFeN catalyst has a relatively smaller Tafel slope of 58.6 mV dec^−1^ in comparison with that of the NiFeN (68.9 mV dec^−1^), NiMoN (82.1 mV dec^−1^), and IrO_2_ electrodes (86.7 mV dec^−1^), verifying its rapid OER catalytic kinetics. We further calculated TOF to assess the intrinsic OER activity of the NiMoN@NiFeN catalyst, which presents a TOF value of 0.09 s^−1^ at an overpotential of 300 mV. This value is not the best among the OER catalysts listed in Supplementary Table 1, but still larger than that of the very good OER catalysts of (Ni,Fe)OOH^12^, Fe_x_Co_1−*x*_OOH^37^, and NiFe-OH/NiFeP^38^. Impressively, our 3D core-shell NiMoN@NiFeN catalyst shows very good durability as well for OER in 1 M KOH electrolyte. As revealed in Fig. 3c, the current densities of 100 and 500 mA cm^−2^ at constant overpotentials show negligible decrease over 48 h OER catalysis, and the CV polarization curves (inset of Fig. 3c) after the stability test remain almost the same as prior to the test. It should be noted that for the stability test at 500 mA cm^−2^, the current density slightly decreases from 499.5 to 480.9 mA cm^−2^ with a degradation rate of 0.775 mA cm^−2 ^h^−1^, which is mainly attributed to the strong adsorption of bubbles blocking the active sites. Moreover, SEM images after OER stability tests (Supplementary Fig. 11) demonstrate the high integrity of the 3D core-shell nanostructures of the NiMoN@NiFeN catalyst. Thus, the long-term robustness mostly originates from its integral 3D core-shell nanostructure with different levels of porosity, which benefits rapid gaseous product release, and the strong adhesion between the TMN catalysts and the Ni foam substrate. To investigate the origins of promoted OER activity in the NiMoN@NiFeN catalyst, we calculated the electrochemical active surface area (ECSA) for the different catalysts by double-layer capacitance (*C*_dl_) from their CV curves (Supplementary Fig. 12)^39^. Clearly, the *C*_dl_ values of the NiMoN and NiMoN@NiFeN catalysts are as large as 188.3 and 238.7 mF cm^−2^ (Supplementary Fig. 13), respectively, which are nearly 2.9 and 3.6 times that of the pure NiFeN nanoparticles (65.4 mF cm^−2^), respectively, demonstrating the highly improved ECSA and the increased number of active sites achieved by decorating NiFeN nanoparticles on the NiMoN nanorods to form a 3D core-shell nanoarchitecture, which benefits seawater adsorption and offers rich active sites for catalytic reactions^40,41^. We further normalized current density by the ECSA, and the NiMoN@NiFeN catalyst still shows better OER activity than that of NiFeN (Supplementary Fig. 14), indicating that factors other than the ECSA also contribute to the enhanced OER activity. For the NiMoN@NiFeN core-shell catalyst, the highly conductive core of NiMoN nanorods and the robust contact between the NiFeN nanoparticles and NiMoN nanorods facilitate the charge transfer between the catalyst and electrolyte, as manifested by the results from electrochemical impedance spectroscopy (EIS, Supplementary Fig. 15), which show that the charge-transfer resistance (*R*_ct_) of this 3D core-shell electrode is only 1.0 Ω, significantly smaller than 9.6 Ω for NiFeN. Additionally, the NiMoN catalyst also has a small *R*_ct_ of 1.7 Ω, confirming its good electronic conductivity and fast charge transfer. Hence, the rational design of 3D core-shell TMN catalysts offers a large surface area and efficient charge transfer, both of which contribute to the improved OER activity.

To seek a good HER catalyst to combine with our NiMoN@NiFeN catalyst for overall seawater splitting, we tested the HER performance of different catalysts, including the benchmark Pt/C on Ni foam, in 1 M KOH in freshwater. Strikingly, both the NiMoN@NiFeN and NiMoN catalysts exhibit exceptional HER activity (Fig. 3d) that is even better than that of the benchmark Pt/C catalyst, especially the NiMoN catalyst, which requires very low overpotentials of 56 and 127 mV for current densities of 100 and 500 mA cm^−2^, respectively. The overpotentials required to achieve the same current densities by our NiMoN@NiFeN catalyst (84 and 180 mV) are slightly higher, but superior to those needed for the Pt/C (96 and 252 mV) and NiFeN (205 and 299 mV) catalysts. NiMoN has been demonstrated to be an efficient HER catalyst in alkaline media because of its excellent electronic conductivity and low adsorption free energy of H*^24,42,43^. Fig. 3e reveals that the NiMoN catalyst also exhibits a much smaller Tafel slope of 45.6 mV dec^−1^ in comparison to the other catalysts measured. Moreover, the NiMoN catalyst shows good stability at current densities of 100 and 500 mA cm^−2^ over 48 h HER testing (Fig. 3f). Therefore, our NiMoN@NiFeN and NiMoN catalysts are highly active and robust for OER and HER, respectively, during freshwater electrolysis in alkaline media.

We then studied the OER and HER activity in an alkaline simulated seawater electrolyte (1 M KOH + 0.5 M NaCl). As shown in Fig. 3g, the 3D core-shell NiMoN@NiFeN catalyst still exhibits outstanding catalytic activity for OER, requiring overpotentials of 286 and 347 mV to achieve current densities of 100 and 500 mA cm^−2^, respectively. This performance is very close to that in the 1 M KOH electrolyte (Fig. 3g), suggesting selective OER in the alkaline adjusted salty water. We also collected natural seawater from Galveston Bay near Houston, Texas, USA (Supplementary Fig. 16) and prepared an alkaline natural seawater electrolyte (1 M KOH + Seawater), in which the OER activity of the NiMoN@NiFeN catalyst shows only slight decay compared with that in the other two electrolytes (Fig. 3g). The slight decrease in activity may be due to some insoluble precipitates [*e.g*., Mg(OH)_2_ and Ca(OH)_2_] covering the surface of the electrode, and thus burying some surface active sites (Supplementary Figs. 17 and 18). Even so, the NiMoN@NiFeN catalyst still delivers current densities of 100 and 500 mA cm^−2^ at small overpotentials of 307 and 369 mV, respectively, in the alkaline natural seawater electrolyte (Fig. 3h). In addition, at an even larger current density of 1000 mA cm^−2^, the demanded overpotential is only 398 mV, which is well below the 490 mV overpotential required to trigger chloride oxidation to hypochlorite. Moreover, this overpotential is also much lower than that of any of the other reported non-precious OER catalysts in alkaline adjusted salty water (Supplementary Table 2). The HER catalyst of NiMoN also exhibits excellent activity in both the alkaline simulated and natural seawater electrolytes (Fig. 3g). To deliver current densities of 100, 500, and 1000 mA cm^−2^ in the alkaline natural seawater, the required overpotentials are as low as 82, 160, and 218 mV, respectively (Fig. 3h). Consequently, our NiMoN@NiFeN and NiMoN catalysts are not only efficient for freshwater electrolysis, but also highly active for alkaline seawater splitting.

### Overall seawater splitting

**Fig. 4:**
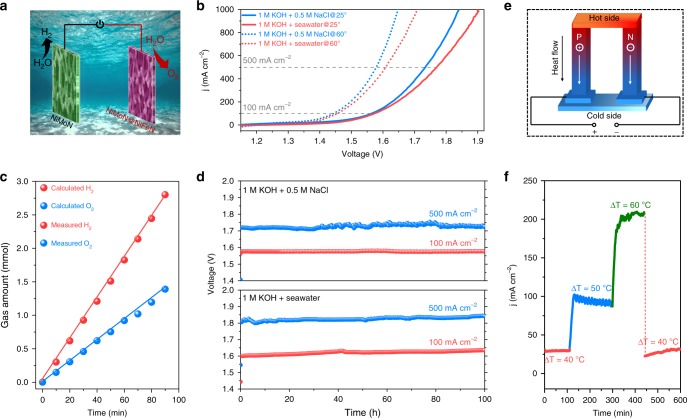
Overall seawater splitting performance. **a** Schematic illustration of an overall seawater splitting electrolyzer with NiMoN and NiMoN@NiFeN as the cathode and anode, respectively. **b** Polarization curves after iR compensation of NiMoN and NiMoN@NiFeN coupled catalysts in a two-electrode electrolyzer tested in alkaline simulated (1 M KOH + 0.5 M NaCl, resistance: ~1.1 Ω) and natural seawater (1 M KOH + Seawater, resistance: ~1.2 Ω) electrolytes under different temperatures. **c** Comparison between the amount of collected and theoretical gaseous products (H_2_ and O_2_) by the two-electrode electrolyzer at a constant current density of 100 mA cm^−2^ in 1 M KOH + 0.5 M NaCl at 25 °C. **d** Durability tests of the electrolyzer at constant current densities of 100 and 500 mA cm^−2^ in different electrolytes at 25 °C. **e** Schematic illustration of the principle for power generation between the hot and cold sides of a TE device. **f** Real-time dynamics of current densities for the electrolyzer in 1 M KOH + 0.5 M NaCl at 25 °C driven by a TE device when the temperature gradient (Δ*T*) between its hot and cold sides is 40, 50, 60, and 40 °C

Considering the outstanding catalytic performance of both the NiMoN@NiFeN and NiMoN catalysts, we further investigated the overall seawater splitting performance by integrating the two catalysts into a two-electrode alkaline electrolyzer (without a diaphragm or membrane), in which NiMoN@NiFeN is used as the anode for OER and NiMoN as the cathode for HER (Fig. 4a). Remarkably, this electrolyzer shows excellent overall seawater splitting activity in both the alkaline simulated and natural seawater electrolytes. As displayed in Fig. 4b, at room temperature (25 °C), the cell voltages needed to produce a current density of 100 mA cm^−2^ are as low as 1.564 and 1.581 V in 1 M KOH + 0.5 M NaCl and 1 M KOH + Seawater electrolytes, respectively. In particular, our electrolyzer can generate extremely large current densities of 500 and 1000 mA cm^−2^ at 1.735 and 1.841 V, respectively, in 1 M KOH + 0.5 M NaCl electrolyte, which is slightly better than the recently reported anion exchange membrane (AEM) based electrolyzer in an alkaline simulated seawater electrolyte^44^. Even in the alkaline natural seawater, the cell voltages for the corresponding current densities are only 1.774 and 1.901 V. Such performance even outperforms that of most non-noble metal catalysts for alkaline freshwater splitting, as well as that of the benchmark of Pt/C and IrO_2_ catalysts in 1 M KOH^15^. To boost the industrial applications of this electrolyzer, the cell voltages are further decreased to 1.454, 1.608, and 1.709 V for current densities of 100, 500, and 1000 mA cm^−2^, respectively, in 1 M KOH + Seawater electrolyte by heating the electrolyte to 60 °C, which can be easily achieved by employing a solar thermal hot water system. These values represent the current record-high performance indices for overall alkaline seawater splitting. The overall seawater splitting performance without iR compensation was also tested in 1 M KOH + Seawater at 25 °C for comparison (Supplementary Fig. 19), and was found to be worse than that with iR compensation. We attempted to split pure natural seawater as well, but the performance is unsatisfactory due to the low ionic conductivity and strong corrosiveness of the natural seawater (Supplementary Fig. 20). We then evaluated the Faradaic efficiency of the electrolyzer in 1 M KOH + 0.5 M NaCl at room temperature by collecting the evolved gaseous products over the cathode and anode (Supplementary Fig. 21). As shown in Fig. 4c, only H_2_ and O_2_ gases with a molar ratio close to 2:1 are detected, and the Faradaic efficiency is determined to be around 97.8% during seawater electrolysis, demonstrating the high selectivity of OER on the anode.

The operating durability is also a very important metric to assess the performance of an electrolyzer. As shown in Fig. 4d, this electrolyzer can retain outstanding overall seawater splitting performance with no noticeable degradation over 100 h operation at a constant current density of 100 mA cm^−2^ in both the alkaline simulated and natural seawater electrolytes. More importantly, the voltage needed to achieve a very large current density of 500 mA cm^−2^ also shows very little increase (<10%) after 100 h water electrolysis in either of the two electrolytes (Fig. 4d), verifying the superior durability of this electrolyzer. The anode of the NiMoN@NiFeN catalyst further demonstrates good structural integrity after long-term seawater electrolysis (Supplementary Fig. 22). In addition, the electrolyzer exhibits very good activity and stability (over 600 h electrolysis) for overall seawater splitting in a very harsh condition of 6 M KOH + Seawater (Supplementary Fig. 23), demonstrating its great potential for large-scale applications. Given its excellent catalytic performance, this electrolyzer can be easily actuated by a 1.5 V AA battery (Supplementary Fig. 24). Moreover, we also demonstrated the harvesting of waste heat, the major energy loss in various activities and device operations, by our seawater electrolyzer powered with a commercial TE device that directly coverts heat into electricity (Fig. 4e)^45^. As shown in Fig. 4f, when the temperature gradient between the hot and cold sides of the TE module is 40, 50, and 60 °C, the corresponding output voltage can expeditiously drive the electrolyzer for stable delivery of current density of 30, 100, and 200 mA cm^−2^, respectively. Even when the temperature gradient through the TE module is decreased to 40 °C, the electrolyzer can still supply a current density of ~30 mA cm^−2^ with good recyclability, suggesting that we can efficiently use the waste heat to produce H_2_ fuel by the electrolysis of seawater.

### Active sites for oxygen evolution catalysis

**Fig. 5:**
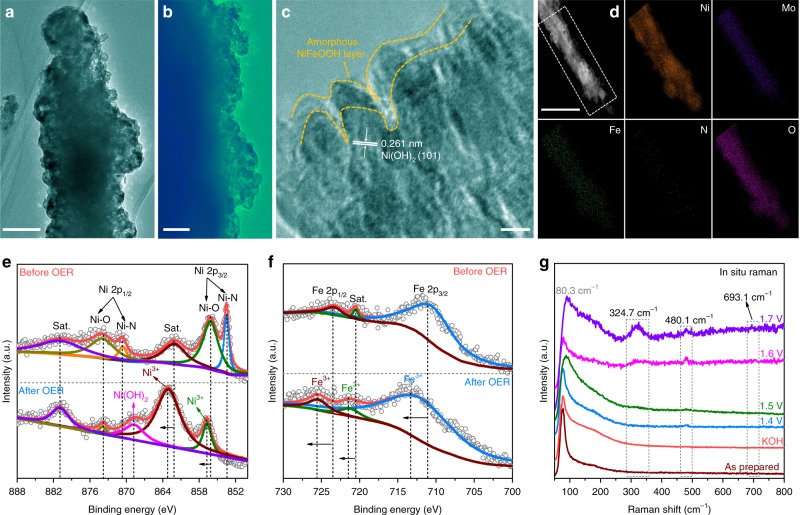
Material characterization to study the OER active sites. **a**, **b** TEM images of NiMoN@NiFeN core-shell nanorods at different magnifications after OER tests. **c** HRTEM image, and **d** DF-STEM image and corresponding elemental mapping of the NiMoN@NiFeN catalyst after OER tests. Scale bars: **a** 500 nm; **b** 50 nm; **c** 5 nm; **d** 1 µm. High-resolution XPS of (**e**), Ni 2p and (**f**), Fe 2p of NiMoN@NiFeN after OER tests in comparison with those before OER tests. **g** In situ Raman spectra of the NiMoN@NiFeN catalyst at various potentials for the OER process

To gain a deeper insight into the real catalytic active sites for the extraordinary OER activity of the NiMoN@NiFeN catalyst, we further studied its nanostructure, surface composition, and chemical state during and after OER tests. The TEM image in Fig. 5a shows that the 3D core-shell nanostructure of NiMoN@NiFeN is intact after OER tests, which is consistent with the SEM results (Supplementary Fig. 11). The TEM image in Fig. 5b reveals that many nanoparticles are closely attached on the nanorod, and there seems to be some very thin layers on the nanoparticle surface. The HRTEM image in Fig. 5c confirms the existence of thin amorphous layers and Ni(OH)_2_. We suspect that the thin layers are in situ generated amorphous NiFe oxides and NiFe oxy(hydroxides), which have been verified by elemental mapping and XPS analyses following OER testing. Figure 5d displays the DF-STEM and corresponding elemental mapping images, which show the absence of N and the increased O content on the NiMoN@NiFeN surface after OER due to the intense oxidation process. The high-resolution XPS of N 1s (Supplementary Fig. 25) also corroborates this point (the surface N content in the NiMoN@NiFeN catalyst was reduced from 10.3% in the fresh sample to 0.36% after OER). For the high-resolution XPS of Ni 2p (Fig. 5e), the two peaks attributed to Ni-N species at 853.4 and 870.8 eV also disappear after OER because of surface oxidation. A new peak at 868.9 eV, which is assigned to Ni(OH)_2_, shows up,. Besides, the two peaks at 856.3 (Ni-O) and 862.0 eV (Sat.) positively shift toward higher binding energy, which is also observed in the XPS of Fe 2p (Fig. 5f), indicating the oxidation of Ni^2+^ and Fe^2+^ to the higher valence states of Ni^3+^ and Fe^3+^ (Supplementary Fig. 26), respectively, resulting from the formation of NiFe oxides/oxy(hydroxides)^46–48^. The O 1s XPS (Supplementary Fig. 27) also proves the increased valence states of Ni^2+^ and Fe^2+^ after OER, as well as showing the appearance of Fe-OH from the NiFe oxy(hydroxides), which can be seen from the negative shift of the main peaks at 531.9 and 530.1 eV and the appearance of a new peak at 532.3 eV^49^. To confirm the formation of NiFe oxides/oxy(hydroxides), we further performed in situ Raman measurements (Supplementary Fig. 28) to elucidate the real-time evolution of the NiMoN@NiFeN catalyst during the OER process. As the results in Fig. 5g show, the spectrum for the as-prepared NiMoN@NiFeN exhibits a sharp and broad peak at around 80.3 cm^−1^, which is probably due to the metal-N stretching modes. The transformation into NiOOH starts at 1.4 V according to a new Raman band located at 480.1 cm^−1^ ^12^. When the potential reaches to 1.6 and 1.7 V, two additional Raman bands are generated. The one located at 324.7 cm^−1^ is assigned to the Fe-O vibrations in Fe_2_O_3_^50^, and the other at 693.1 cm^−1^ belongs to the Fe-O vibrations in amorphous FeOOH^51^. Therefore, by combining these results with the XPS results, we conclude that thin amorphous layers of NiFe oxide and NiFe oxy(hydroxide) are evolved from the NiFeN nanoparticles at the surface during OER electrocatalysis, and that these serve as the real active sites participating in the OER process. The formation of a metal nitride-metal oxide/oxy(hydroxide) core-shell structure may also facilitate electron transfer from the NiFeN core to the oxidized species (Supplementary Fig. 29). This observation is consistent with the results of other reported OER catalysts, including metal selenides and phosphides^14,46^. However, the structure of the in situ formed NiFe oxides/oxy(hydroxides) is different from that of the (Ni,Fe)OOH thin-film catalyst reported by Zhou et al.^12^, which undergoes a rapid self-reconstruction due to the partial dissolution of FeOOH in KOH solution, forming amorphous NiOOH nanoarrays mixed with a small amount of FeOOH nanoparticles after OER. Notably, such in situ generated amorphous NiFe oxide and NiFe oxy(hydroxide) layers also play a positive role in improving the resistance to corrosion by chloride anions in seawater (Supplementary Fig. 30), which contributes to the superior stability during seawater electrolysis.

## Discussion

In summary, we have developed a 3D core-shell OER catalyst of NiMoN@NiFeN for active and stable alkaline seawater splitting. The interior NiMoN nanorods are highly conductive and afford a large surface area, which ensure efficient charge transfer and numerous active sites. The outer NiFeN nanoparticles in situ evolve thin amorphous layers of NiFe oxide and NiFe oxy(hydroxide) during OER catalysis, which are not only responsible for the selective OER activity, but also beneficial for the corrosion resistance to chloride anions in seawater. At the same time, the 3D core-shell nanostructures with multiple levels of porosity are favorable for seawater diffusion and H_2_/O_2_ gases releasing. Thus, this OER catalyst requires very low overpotentials of 369 and 398 mV to deliver large current densities of 500 and 1000 mA cm^−2^, respectively, in alkaline natural seawater at 25 °C. Additionally, by pairing it with another efficient HER catalyst of NiMoN, we assembled an outstanding water electrolyzer for overall seawater splitting, which outputs current densities of 500 and 1000 mA cm^−2^ at record low voltages of 1.608 and 1.709 V, respectively, in alkaline natural seawater at 60 °C. The electrolyzer also shows excellent durability at current densities of 100 and 500 mA cm^−2^ during up to 100 h alkaline seawater electrolysis. This discovery represents a significant step in the development of a robust and active catalyst to utilize the world’s abundant seawater feedstock for large-scale hydrogen production by renewable energy sources.

## Methods

### Chemicals

Ethanol (C_2_H_5_OH, Decon Labs, Inc.), ammonium heptmolybdate [(NH_4_)_6_Mo_7_O_24_·4H_2_O, 98%, Sigma-Aldrich], nickel(II) nitrate hexahydrate (Ni(NO_3_)_2_·6H_2_O, 98%, Sigma-Aldrich), iron (III) nitrate hexahydrate (Fe(NO_3_)_3_·9H_2_O, 98%, Sigma-Aldrich), N, N Dimethylformamide [DMF, (CH_3_)_2_NC(O)H, anhydrous, 99.8%, Sigma-Aldrich], platinum powder (Pt, nominally 20% on carbon black, Alfa Aesar), iridium oxide powder (IrO2, 99%, Alfa Aesar), Nafion (117 solution, 5% wt, Sigma-Aldrich), sodium chloride (NaCl, Fisher Chemical), potassium hydroxide (KOH, 50% w/v, Alfa Aesar), and Ni foam (thickness: 1.6 mm, porosity: ~95%) were used as received. Deionized (DI) water (resistivity: 18.3 MΩ·cm) was used for the preparation of all aqueous solutions.

### Synthesis of NiMoO_4_ nanorods on Ni foam

NiMoO_4_ nanorods were synthesized on nickel foam through a hydrothermal method^52^. A piece of commercial Ni foam (2 × 5 cm^2^) was cleaned by ultrasonication with ethanol and DI water for several minutes, and the substrate was then transferred into a polyphenyl (PPL)-lined stainless-steel autoclave (100 ml) containing a homogenous solution of Ni(NO_3_)_2_·6H_2_O (0.04 M) and (NH_4_)_6_Mo_7_O_24_·4H_2_O (0.01 M) in 50 ml H_2_O. Afterward, the autoclave was sealed and maintained at 150 °C for 6 h. The sample was then taken out and washed with DI water and ethanol several times before being fully dried at 60 °C overnight under vacuum.

### Synthesis of NiMoN@NiFeN core-shell nanorods

The metal nitrides were synthesized by one-step nitridation of the NiMoO_4_ nanorods in a tube furnace. For the synthesis of NiMoN nanorods, a piece of NiMoO_4_/Ni foam (~1 cm^2^) was placed at the middle of a tube furnace and thermal nitridation was conducted at 500 °C under a flow of 120 standard cubic centimeters (sccm) NH_3_ and 30 sccm Ar for 1 h. The furnace was then automatically turned off and naturally cooled down to room temperature under Ar atmosphere. For the synthesis of NiMoN@NiFeN core-shell nanorods, a piece of NiMoO_4_/Ni foam (~1 cm^2^) was first soaked in a NiFe precursor ink, which was prepared by dissolving Ni(NO_3_)_2_·6H_2_O and Fe(NO_3_)_3_·9H_2_O with a mole ratio of 1:1 in DMF, and the NiMoO_4_/Ni foam coated with the NiFe precursor ink was then dried at ambient condition. The dried sample was subjected to thermal nitridation under the same conditions as for NiMoN. To study the effect of the NiFeN loading amount on the morphology of the core-shell nanorods, we prepared four different NiMoN@NiFeN core-shell nanorods with different loading amounts of NiFeN by controlling the concentration of Ni and Fe precursors. Specifically, 0.1, 0.25, 0.5, and 0.75 g ml^−1^ concentrations of precursor ink were used. For comparison, pure NiFeN nanoparticles were also prepared on the Ni foam by replacing the NiMoO_4_/Ni foam with Ni foam. The concentration of precursor ink in this case was 0.25 g ml^−1^, and all other synthesis conditions were the same as for NiMoN@NiFeN.

### Preparation of IrO_2_ and Pt/C catalysts on Ni foam

To prepare the IrO_2_ electrode for comparison^53^, 40 mg of IrO_2_ and 60 μL of Nafion were dispersed in 540 μL of ethanol and 400 μL of DI water, and the mixture was ultrasonicated for 30 min. The dispersion was then coated onto a Ni foam substrate, which was dried in air overnight. Pt/C electrodes were obtained by the same method.

### Materials characterization

The morphology and nanostructure of the samples were determined by scanning electron microscopy (SEM, LEO 1525) and transmission electron microscopy (TEM, JEOL 2010F) coupled with energy dispersive X-ray (EDX) spectroscopy. The phase composition of the samples was characterized by X-ray diffraction (PANalytical X’pert PRO diffractometer with a Cu Ka radiation source) and XPS (PHI Quantera XPS) using a PHI Quantera SXM scanning X-ray microprobe.

### Electrochemical tests

The electrochemical performance was tested on an electrochemical station (Gamry, Reference 600). The two half reactions of OER and HER were each carried out at room temperature (~25 °C) in a standard three-electrode system with our prepared sample as the working electrode, a graphite rod as the counter electrode, and a standard Hg/HgO electrode as the reference electrode. Four different electrolytes, including 1 M KOH, 1 M KOH + 0.5 M NaCl, 1 M KOH + Seawater, and natural seawater, were used, and the pH was around 14 except for the natural seawater (pH~7.2). The electrode size is around 1 cm^−2^, and the effective parts in the electrolyte are 0.3 ~ 0.45 cm^−2^ for different samples. Both the anodes (NiMoN@NiFeN) and cathodes (NiMoN) were cycled ~100 times by CV until a stable polarization curve was developed prior to measuring each polarization curve. OER polarization curve measurements were performed by CV at a scan rate of 2 mV s^−1^ and stability tests were carried out under constant overpotentials. HER polarization curve measurements were performed by linear sweep voltammetry (LSV) at a scan rate of 5 mV s^−1^ and stability tests were carried out under constant overpotentials. Electrochemical impedance spectra (EIS) were measured at an overpotential of 270 mV from 0.1 Hz to 100 KHz with an amplitude of 10 mV. For the two-electrode seawater electrolysis, the as-prepared NiMoN@NiFeN and NiMoN catalysts (after CV activation) were used as the anode and cathode, respectively. The polarization curves were measured in different electrolytes at different temperatures (25 and 60 °C), and stability tests were carried out under constant current densities of 100 and 500 mA cm^−2^ at room temperature.

### Turnover frequency (TOF) calculation

The TOF value is calculated from the Eq. (1)^54,55^:

1 $${\mathrm{TOF}} = \frac{{j \ast A \ast {\mathrm{\eta }}}}{{4 \ast F \ast n}}$$

where *j* is the current density at a given overpotential, *A* is the surface area of the electrode, *η* is the OER Faradaic efficiency, *F* is the Faraday constant, and *n* is the total number of active sites on the electrode. To calculate TOF, the most significant challenge is to identify the number of active sites. Since the real active sites for the NiMoN@NiFeN catalyst are in situ formed NiFe oxide and NiFe oxy(hydroxide), we calculated the number of active sites by integrating the Ni redox peaks from the CV curve according to the following Eq. (2)^55^:

2 $$n = \frac{{Q \ast N_A}}{{F \ast R_{{\mathrm{Ni}}/{\mathrm{Fe}}}}}$$

where *Q* is the integration of Ni redox features from the CV curve (Supplementary Fig. 9d), *N*_*A*_ is Avogadro’s constant, *F* is the Faraday constant, and *R*_Ni/Fe_ is the molar ratio of Ni/Fe, assuming that Ni^2+^/Ni^3+^ is a one-electron process. For the NiMoN@NiFeN catalyst at overpotential of 300 mV, *j* = 206 mA cm^−2^, and thus TOF was calculated to be 0.09 s^−1^ at overpotential of 300 mV.

### Gas chromatography measurement

Overall seawater splitting for gas chromatography (GC, GOW-MAC 350 TCD) tests were performed in a gas-tight electrochemical cell with 1 M KOH + 0.5 M NaCl as the electrolyte at room temperature (25 °C). Chronopotentiometry was applied with a constant current density of 100 mA cm^−2^ to maintain oxygen and hydrogen generation. For each measurement over an interval of 10 min, 0.3 µL gas sample was carefully extracted from the sealed cell and injected into the GC instrument using a glass syringe (Hamilton Gastight 1002).

### Overall seawater splitting driven by a TE module

We purchased a commercial TE module from Amazon and used it as a power generator to drive our two-electrode electrolyzer according to our previous work^12^. During the test, the hot side of the TE module was covered by a large flat copper plate, which was in direct contact with a heater on top. The hot-side temperature was maintained relatively constant by tuning the DC power supply to the heater, while the cold-side temperature was controlled by placing it in direct contact with a cooling system, where the water inside was adjusted to remain at a constant temperature. Thus, the TE module generated a relatively stable open circuit voltage between the hot and cold sides. A nano-voltmeter and an ammeter were embedded into the circuit for real-time monitoring of the voltage and current, respectively, between the two electrodes of the water-splitting cell.

## Supplementary information

Supplementary Information

Source data

## Data Availability

The source data underlying Figs. 1i, 2a–f, 3c, f and h, 4c and f, 5e–g, and Supplementary Figs. 2a, 6, 12, 20, 21a, 23a, 25, 26, and 27 are provided as a Source Data file. The other data that support the findings of this work are available from the corresponding authors upon reasonable request.

## Code availability

All plots and data analysis were performed with OriginPro 8.5 software, and will be made available by the corresponding authors upon reasonable request.
