# Supplementary material for: Non-noble metal-nitride based electrocatalysts for high-performance alkaline seawater electrolysis
Source: Nat Commun. 2019 Nov 8;10:5106. doi: 10.1038/s41467-019-13092-7 (PMC6841982; doi:10.1038/s41467-019-13092-7)
Supplement: Supplementary file 1 — Supplementary Information [file 41467_2019_13092_MOESM1_ESM.pdf]

Supplementary Information for

Non-noble metal-nitride based electrocatalysts for high-performance alkaline seawater electrolysis

Yu *et al.*

Supplementary Figures

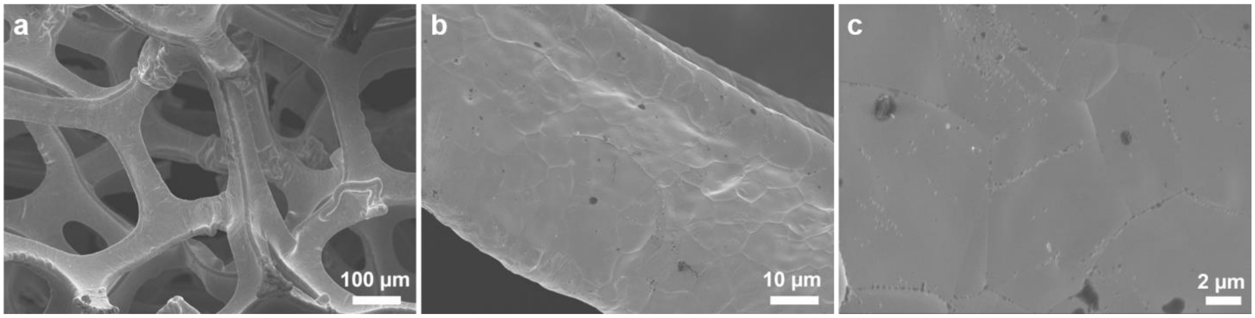

Supplementary Figure 1 | Morphology of Ni foam. SEM images of the commercial Ni foam at (a-c) different magnifications.

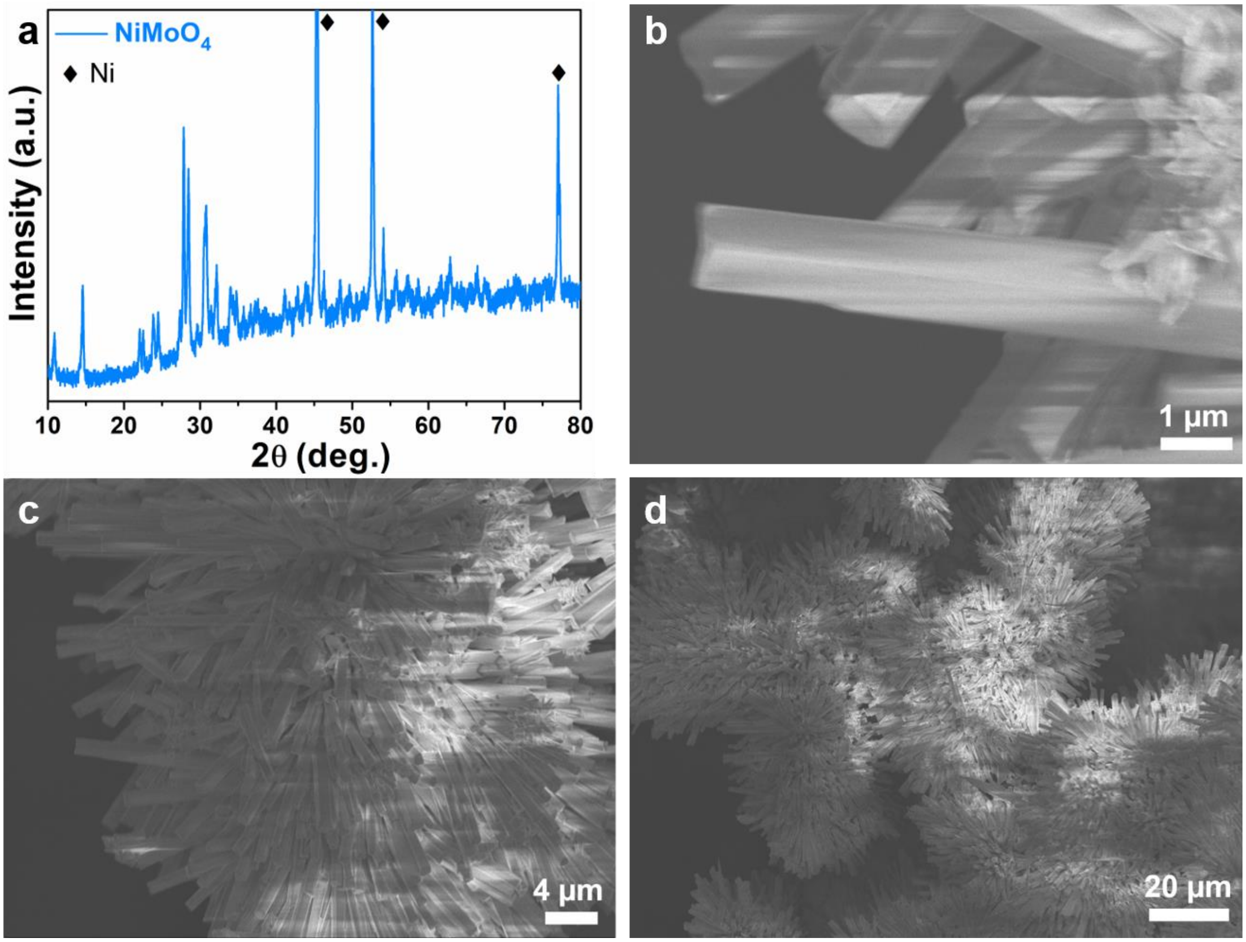

Supplementary Figure 2 | Characterizations of $NiMoO_4$. (a) XRD pattern of $NiMoO_4$ on Ni foam. (b-d) SEM images of $NiMoO_4$ nanorods on Ni foam at different magnifications.

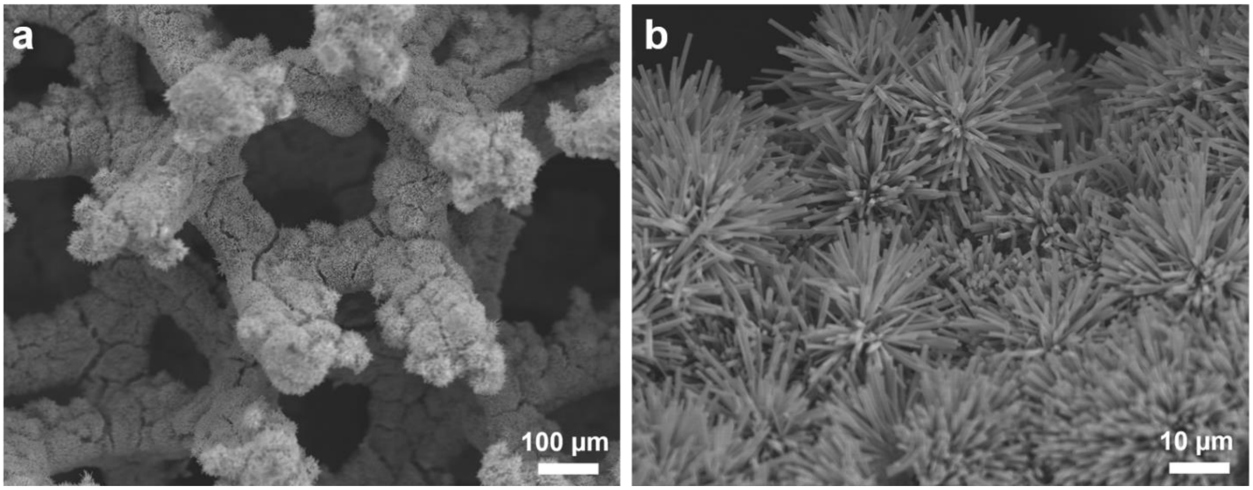

Supplementary Figure 3 | SEM patterns of NiMoN. SEM images of NiMoN nanorods on Ni foam at (a) low and (b) high magnifications.

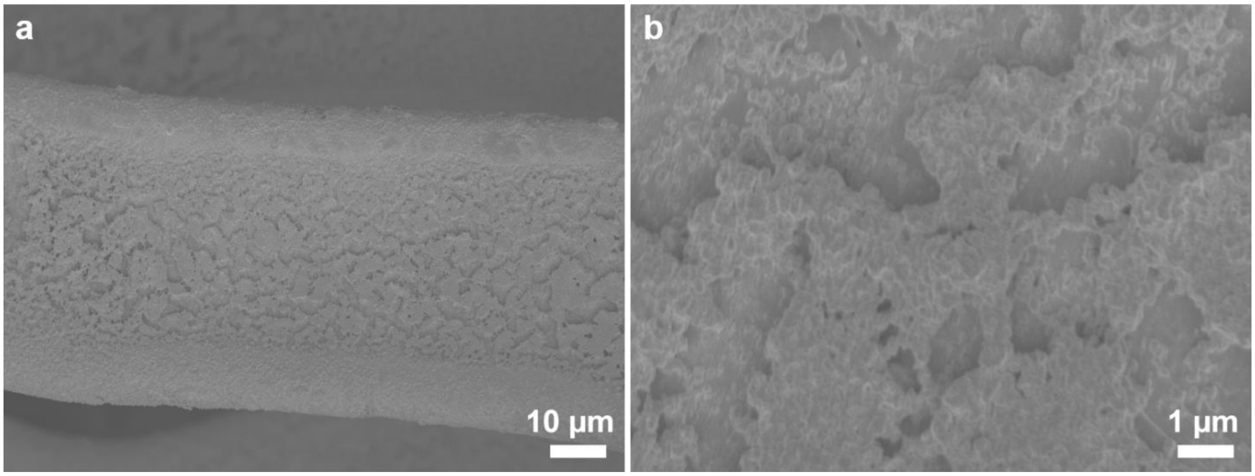

Supplementary Figure 4 | SEM patterns of NiFeN. SEM images of NiFeN nanoparticles on Ni foam at (a) low and (b) high magnifications.

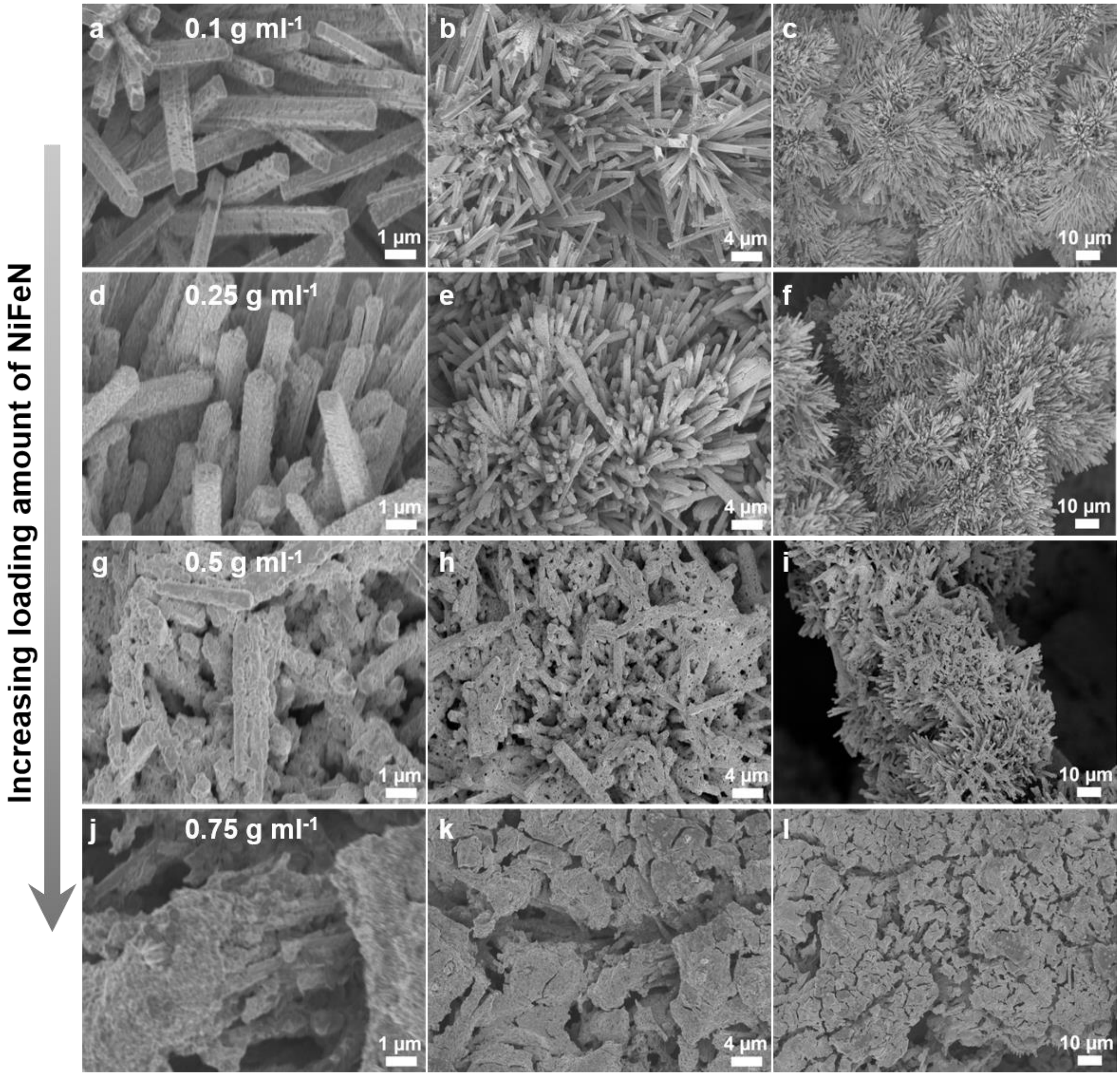

Supplementary Figure 5 | Morphology variation of NiMoN@NiFeN. SEM images at different magnifications of NiMoN@NiFeN core-shell nanorods prepared with different loading amounts of NiFeN nanoparticles by controlling the concentration of NiFe precursors. (a-c) 0.1 g ml^{-1}; (d-f) 0.25 g ml^{-1}; (g-i) 0.5 g ml^{-1}; and (j-l) 0.75 g ml^{-1}.

The surface morphology of the NiMoN@NiFeN core-shell nanorods changes greatly upon varying the concentration of NiFe precursors, which determines the loading amount of NiFeN

nanoparticles. We prepared four different NiMoN@NiFeN samples under precursor ink concentrations of 0.1, 0.25, 0.5, and 0.75 g ml^{-1}, and the corresponding loading mass values of NiFeN nanoparticles were 0.84, 1.27, 1.88, and 2.33 g cm^{-2}, respectively. With a precursor ink concentration of 0.1 g ml^{-1}, some NiFeN nanoparticles are randomly interspersed on the surfaces of the NiMoN nanorods (Supplementary Figs. 5a-5c). When the concentration is 0.25 g ml^{-1}, the entire surfaces of the NiMoN nanorods are uniformly decorated with many NiFeN nanoparticles (Supplementary Figs. 5d-5f), and the large interspace between the neighboring nanorods is well preserved, thus maximizing the active surface area with different levels of porosity. When the concentration is increased to 0.5 g ml^{-1}, the nanoparticles are aggregated on the NiMoN surfaces as well as in the interspaces between the nanorods (Supplementary Figs. 5g-5i). When the concentration is further increased to 0.75 g ml^{-1}, the NiMoN nanorods are almost buried, and the interspaces between the nanosheets are completely filled with the NiFeN nanoparticles (Supplementary Figs. 5j-5l), thereby reducing the surface area. Therefore, the optimal concentration of precursor ink is 0.25 g ml^{-1}.

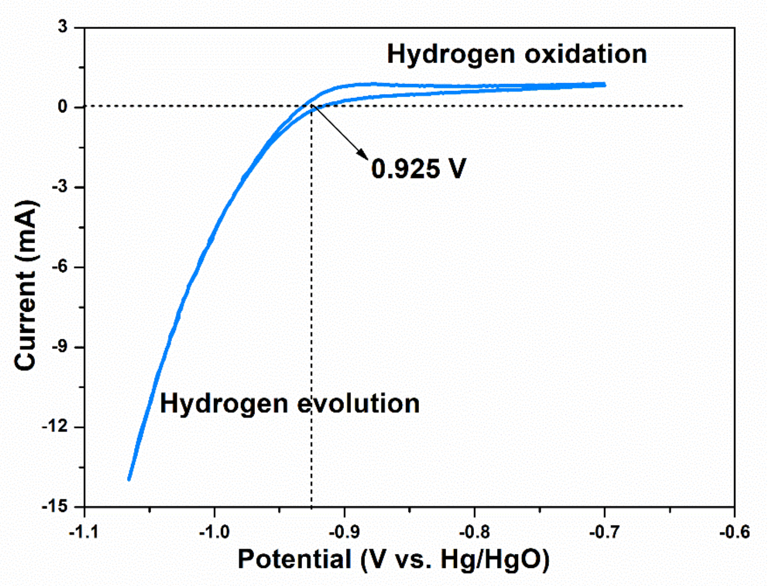

Supplementary Figure 6 | Reference electrode calibration. Calibration of the Hg/HgO reference electrode with respect to RHE in 1 M KOH.

The reference electrode (Hg/HgO) calibration was performed in a high-purity hydrogen-saturated 1 M KOH electrolyte with a Pt foil serving as the working electrode.[1] Cyclic voltammetry (CV) was conducted at a scan rate of 2 mV s^{-1}, and the average of the two potentials at which the current crossed zero was regarded as the thermodynamic potential for the hydrogen electrode reaction. Thus, in 1 M KOH, $E_{RHE} = E_{Hg/HgO} + 0.925$ V. The CV curve is shown in Supplementary Fig. 6.

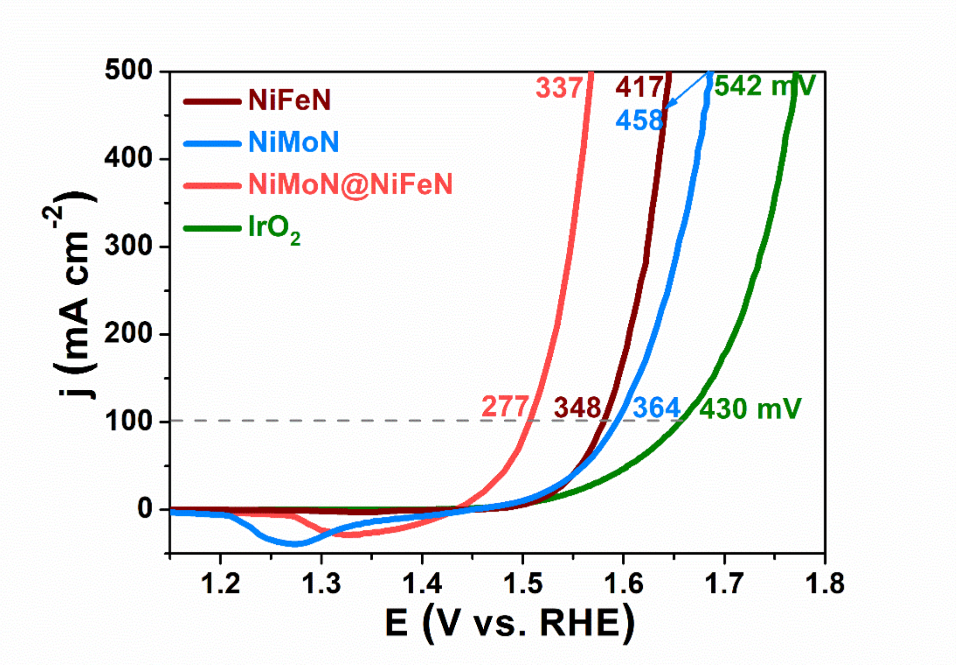

Supplementary Figure 7 | CV backward scan polarization curves. CV backward scan polarization curves of different electrodes tested in 1 M KOH at room temperature.

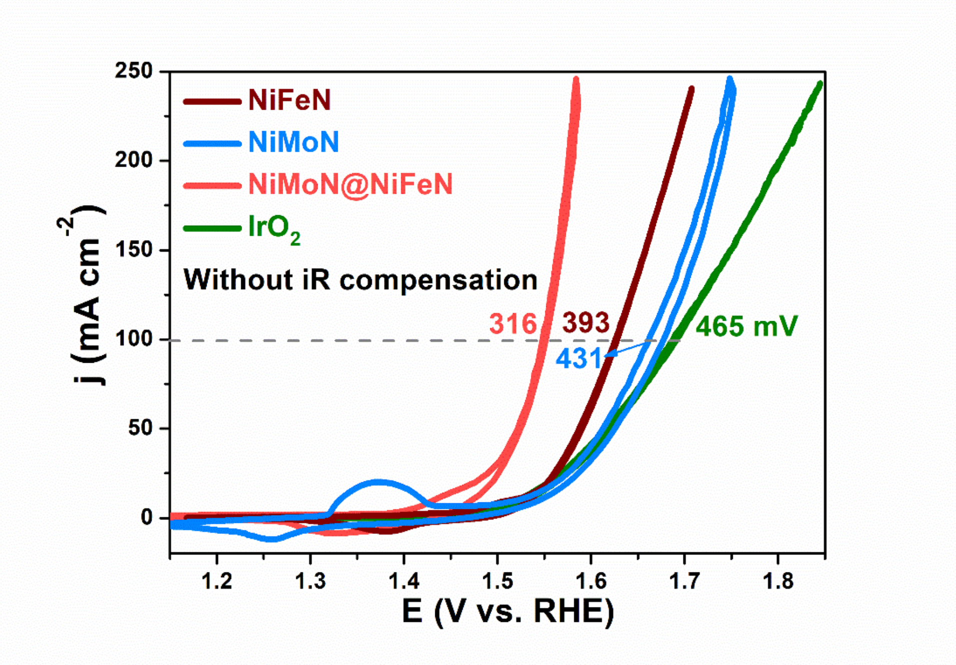

Supplementary Figure 8 | CV polarization curves without iR compensation. OER CV polarization curves of different catalysts tested in 1 M KOH at room temperature without iR compensation.

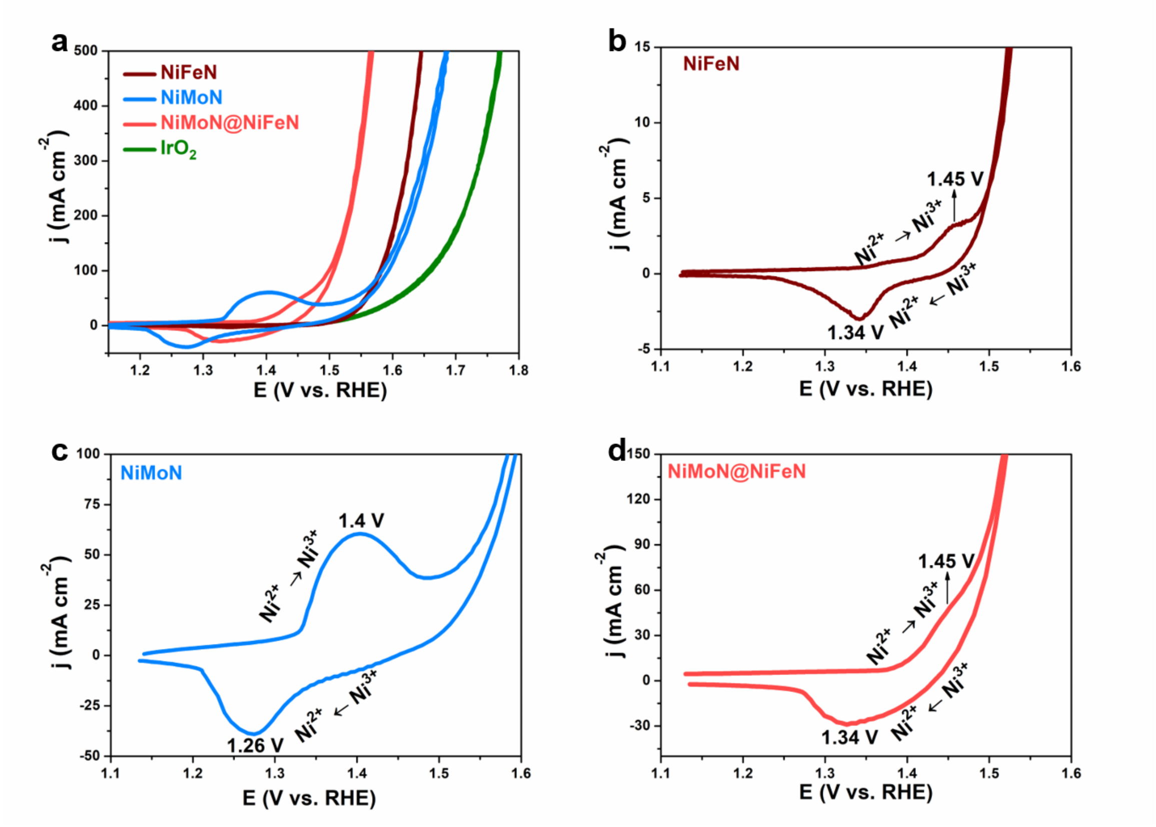

Supplementary Figure 9 | CV polarization curves with iR compensation. (a) OER CV polarization curves of different catalysts tested in 1 M KOH at room temperature with iR compensation. Partial CV curves of (b) NiFeN, (c) NiMoN, and (d) NiMoN@NiFeN from (a) selected to study the redox behaviors of the metal-nitride catalysts.

We tested the CV curves for the different catalysts (Supplementary Fig. 9a) and then selected the redox parts in the range of about 1.125 ~ 1.525 V *vs.* RHE to study the redox behaviors of the metal-nitride catalysts. As shown in Supplementary Figs. 9b and 9d, both the NiFeN and NiMoN@NiFeN catalysts exhibit reduction peaks of Ni^{3+} to Ni^{2+} at 1.34 V and oxidation peaks of Ni^{2+} to Ni^{3+} at 1.45 V.[2,3] However, these two peaks shift to lower potentials (1.26 and 1.4 V *vs.* RHE, respectively) for the NiMoN catalyst (Supplementary Fig. 9c). This may be due to the

different accompanying atoms of Fe and Mo, since it has been reported that Mo facilitates oxidation of Ni^{2+} to Ni^{3+} at lower potentials, while Fe appears to stabilize the Ni^{2+} oxidation state.[3,4]

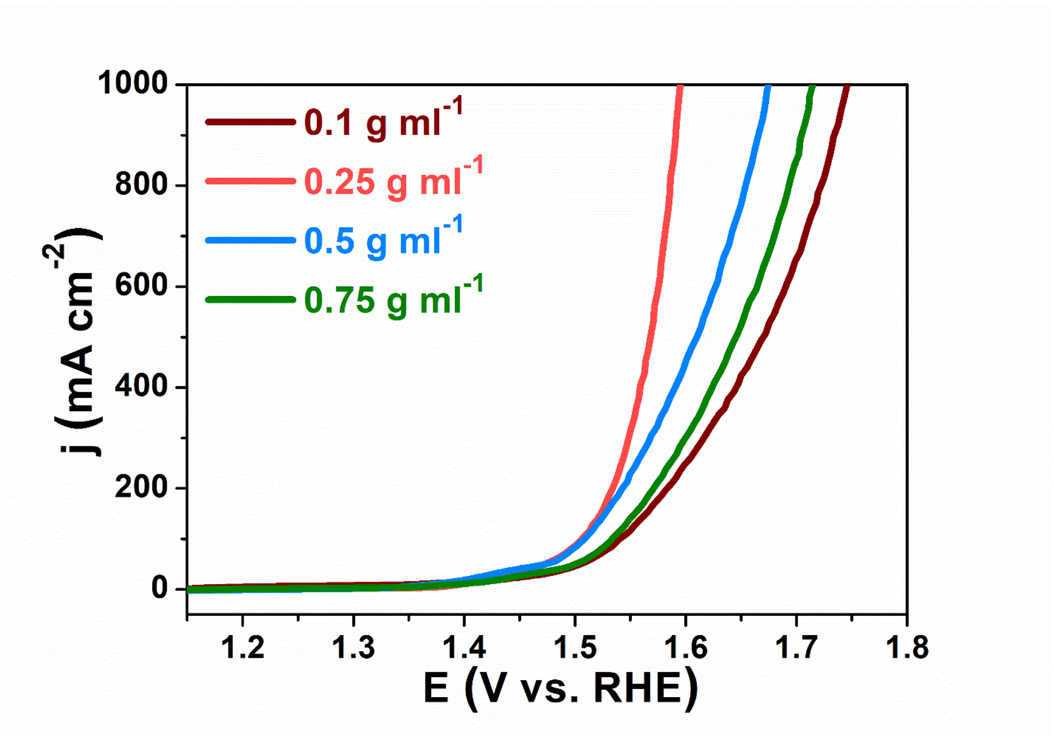

Supplementary Figure 10 | OER activity of different NiMoN@NiFeN catalysts. OER polarization curves (1 M KOH, 25 °C) of different NiMoN@NiFeN catalysts prepared with different loading amounts of NiFeN nanoparticles by controlling the concentration of NiFe precursors.

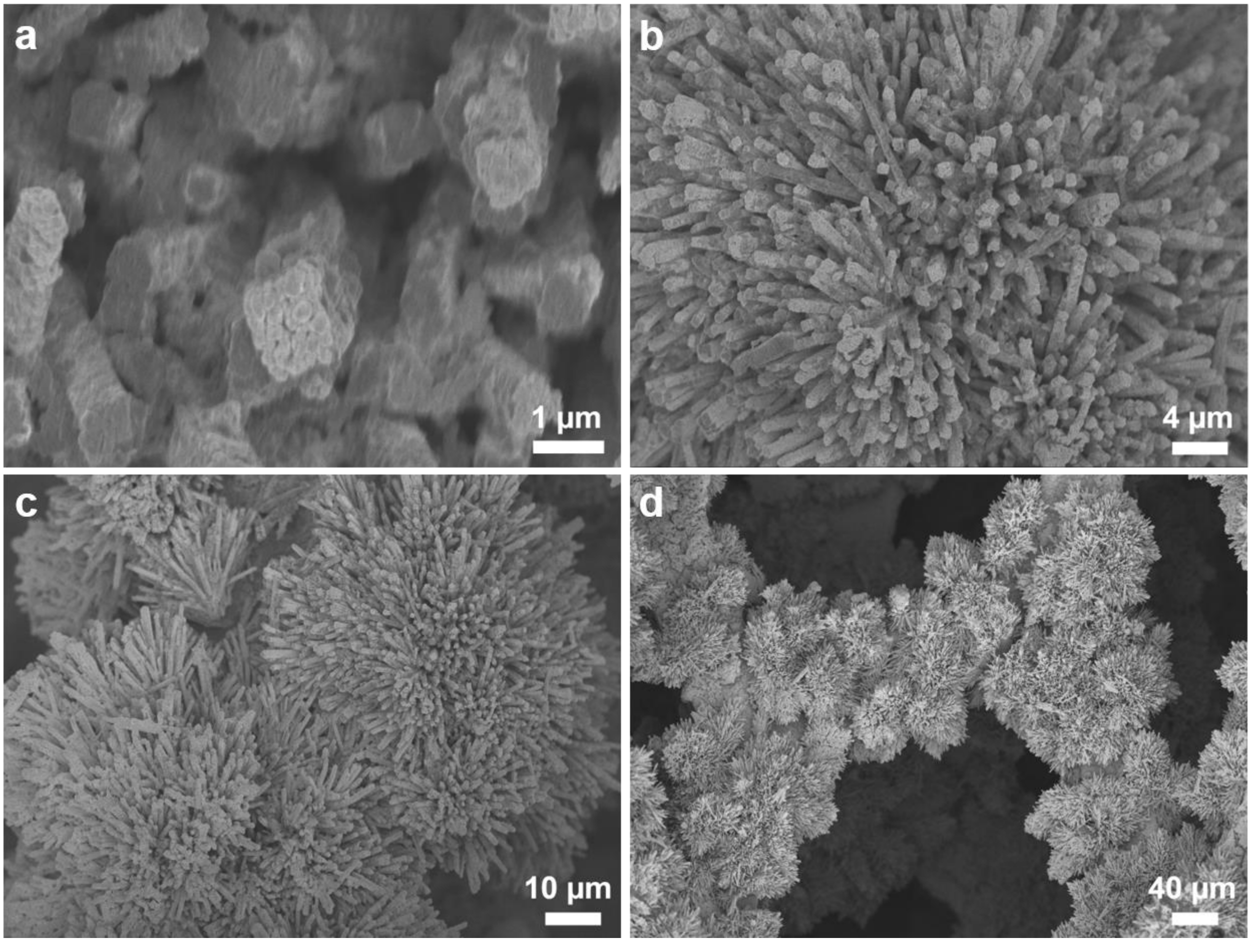

Supplementary Figure 11 | Morphology of NiMoN@NiFeN after OER. SEM images at (a-d) different magnifications of NiMoN@NiFeN core-shell nanorods after OER stability tests.

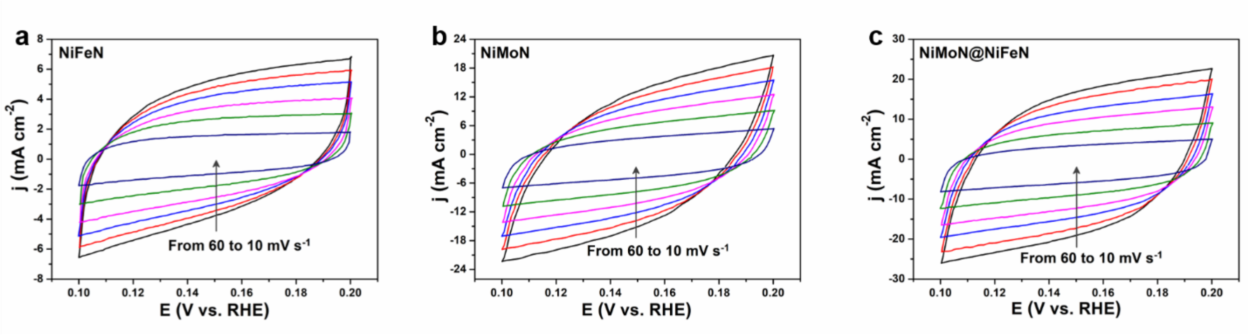

Supplementary Figure 12 | CV curves for calculation of double-layer capacitance. CV curves of (a) NiFeN, (b) NiMoN, and (c) NiMoN@NiFeN at scan rates ranging from 10 mV s^{-1} to 60 mV s^{-1} with an interval point of 10 mV s^{-1}.

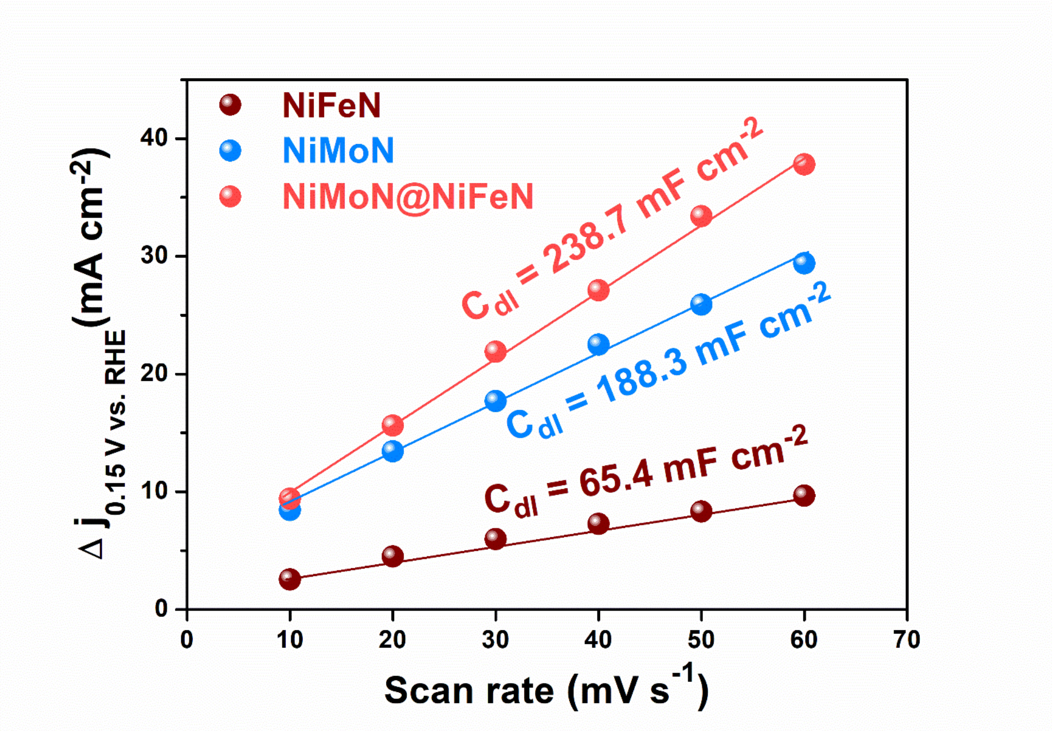

Supplementary Figure 13 | Calculation of double-layer capacitance. Linear fitting of the capacitive currents of the catalysts *vs.* the scan rates to calculate the double-layer capacitance (C_{dl}).

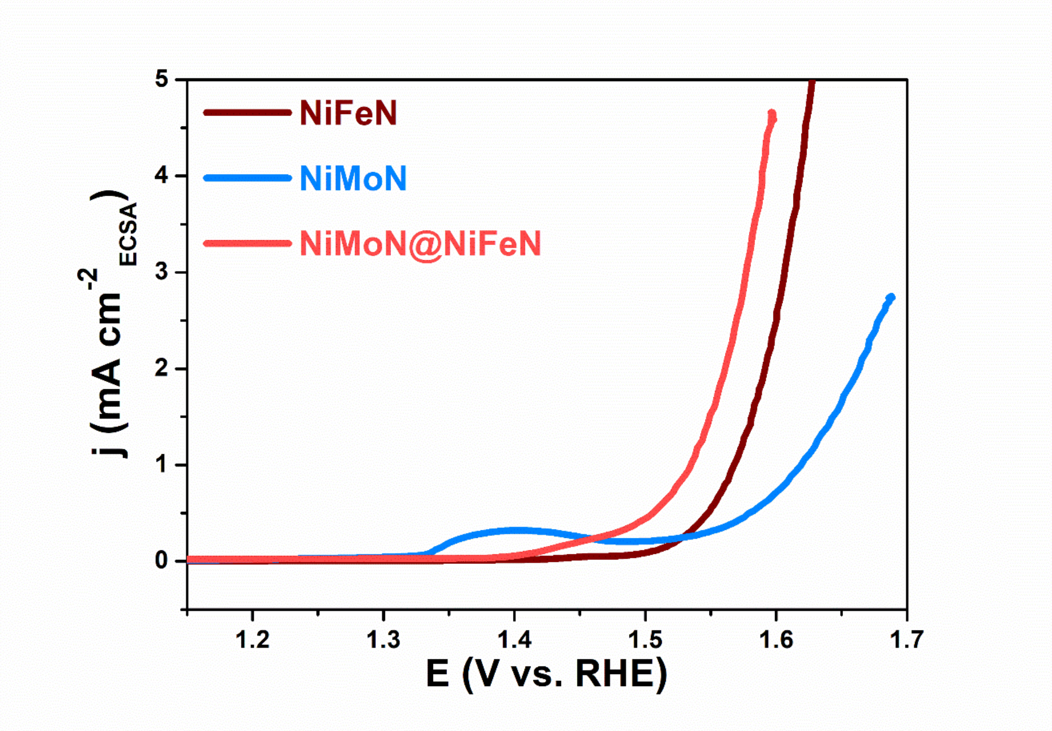

Supplementary Figure 14 | OER activity normalized by ECSA. OER polarization curves in 1 M KOH at 25 °C for different catalysts normalized by the electrochemical active surface area (ECSA).

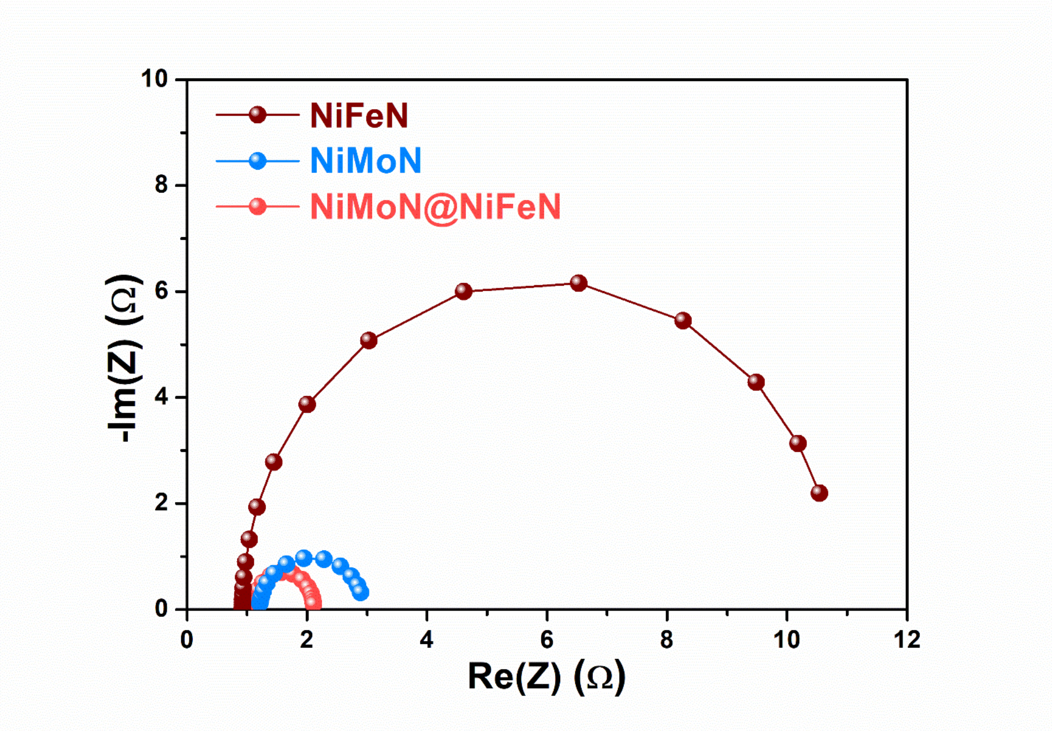

Supplementary Figure 15 | EIS results. EIS Nyquist plots of different catalysts tested at potential of 1.5 V *vs.* RHE.

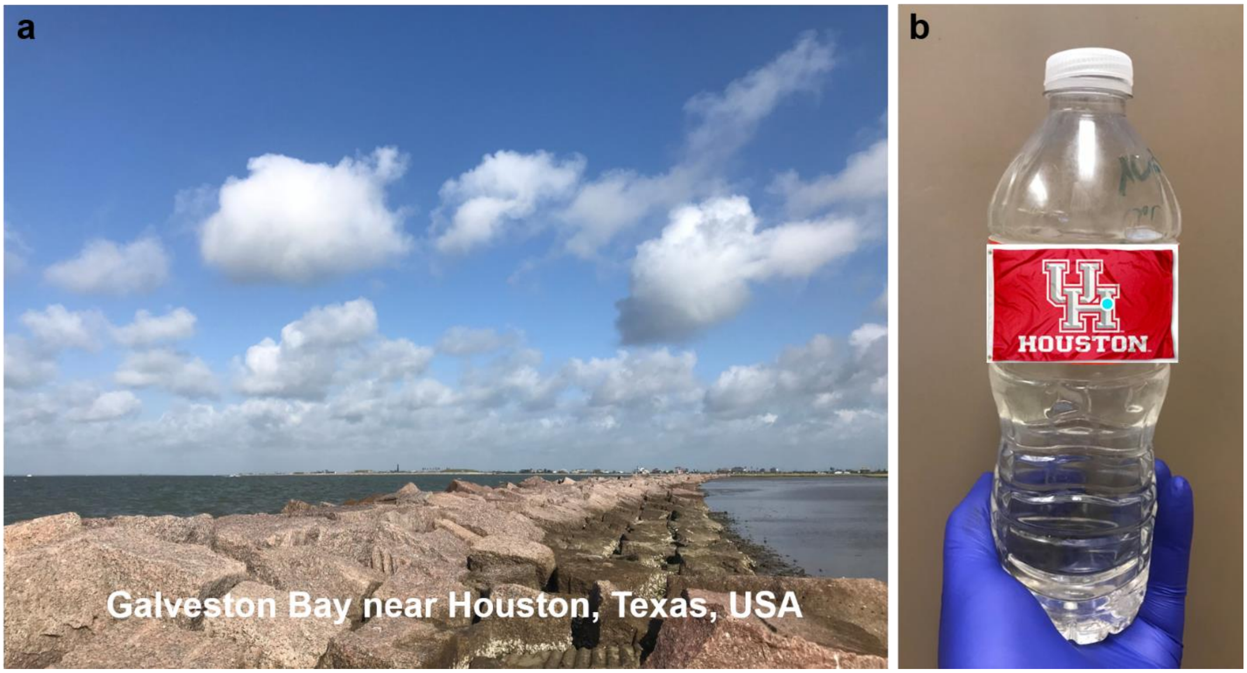

Supplementary Figure 16 | Collection of natural seawater. (a) Photograph of Galveston Bay near Houston, Texas, USA. (b) Photograph of the collected natural seawater (pH around 7.2).

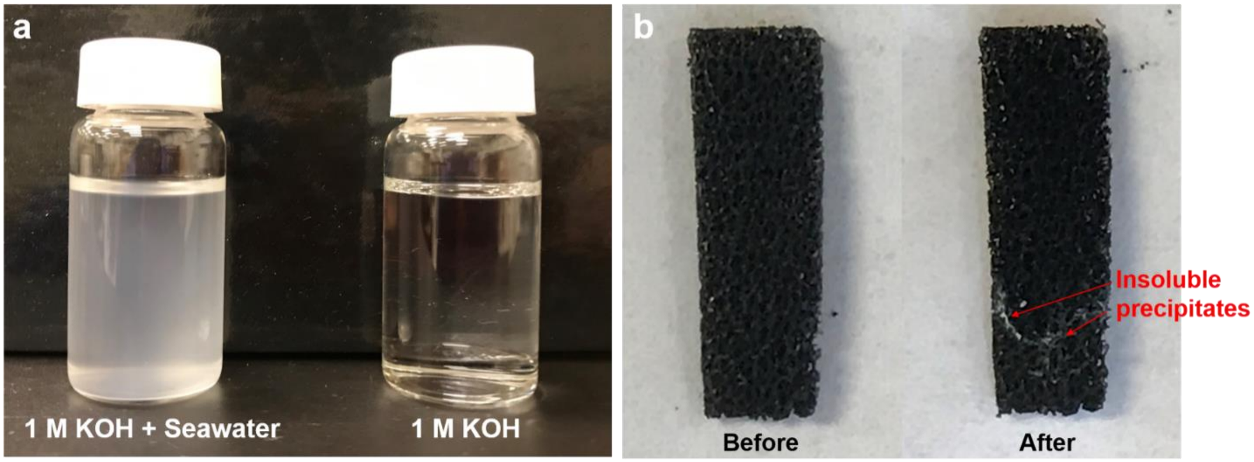

Supplementary Figure 17 | Evidence of insoluble precipitates. Optical images of (a) the two electrolytes, and (b) the NiMoN@NiFeN sample before and after seawater electrolysis.

As shown in the optical images in Supplementary Fig. 17a, the 1 M KOH electrolyte is completely clear and transparent, while the electrolyte of 1 M KOH + Seawater is cloudy with suspended sediments. In Supplementary Fig. 17b, the optical images of the NiMoN@NiFeN sample clearly show that there are some white insoluble precipitates covering the catalyst surface after seawater electrolysis.

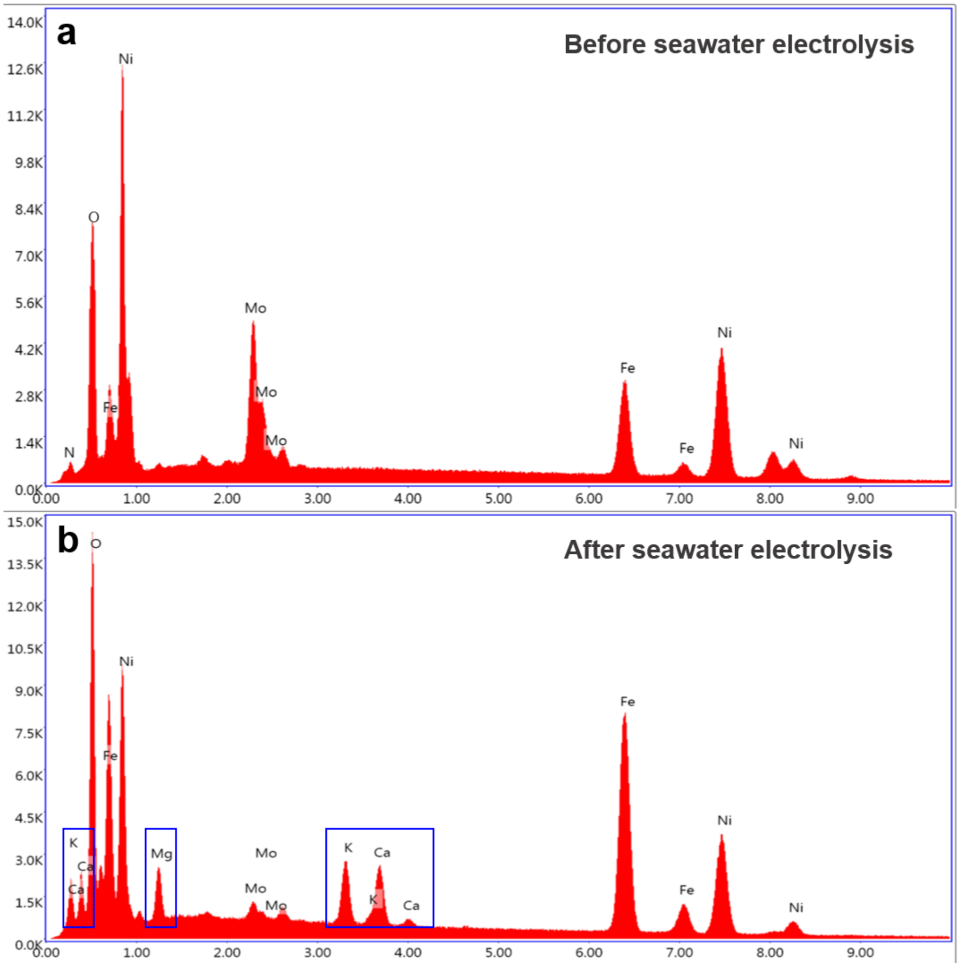

Supplementary Figure 18 | Identification of insoluble precipitates. EDX spectra of the NiMoN@NiFeN catalyst (a) before, and (b) after seawater electrolysis.

As shown in Supplementary Fig. 18, after seawater electrolysis, several new metal elements including Mg, Ca, and K, which come from the seawater, are detected. Therefore, the white insoluble precipitates covering the NiMoN@NiFeN surface (Supplementary Fig. 17b) should be $Mg(OH)_2$ and $Ca(OH)_2$, which bury some surface active sites and weaken the catalytic activity in the seawater electrolyte.

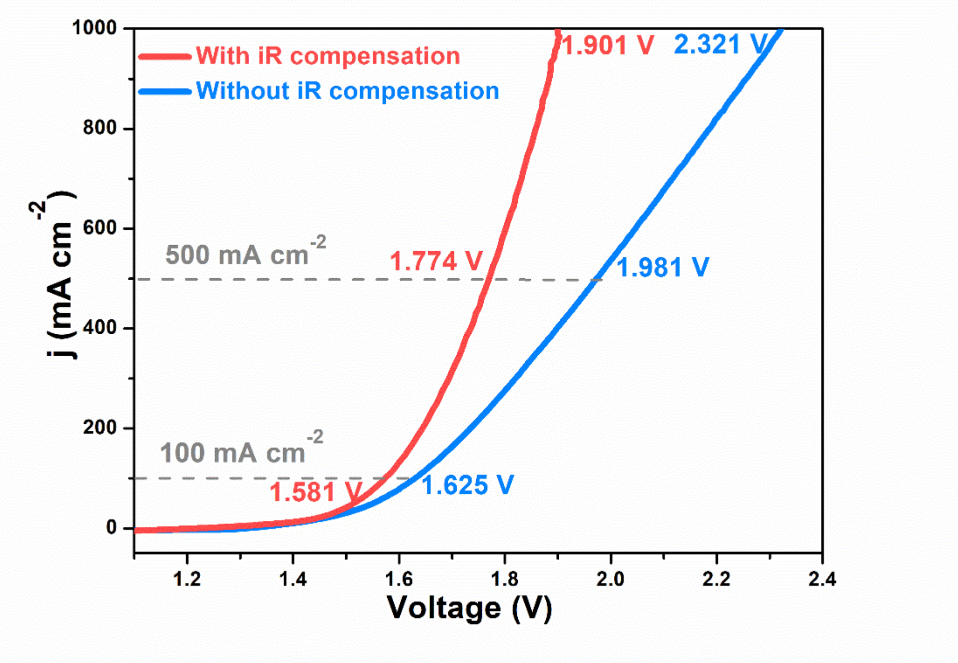

Supplementary Figure 19 | Activity comparison between with iR and without iR compensation. Polarization curves of NiMoN@NiFeN||NiMoN for overall seawater splitting with and without iR compensation. Electrolyte: 1 M KOH + Seawater (resistance: ~1.2 Ω); temperature: 25 °C.

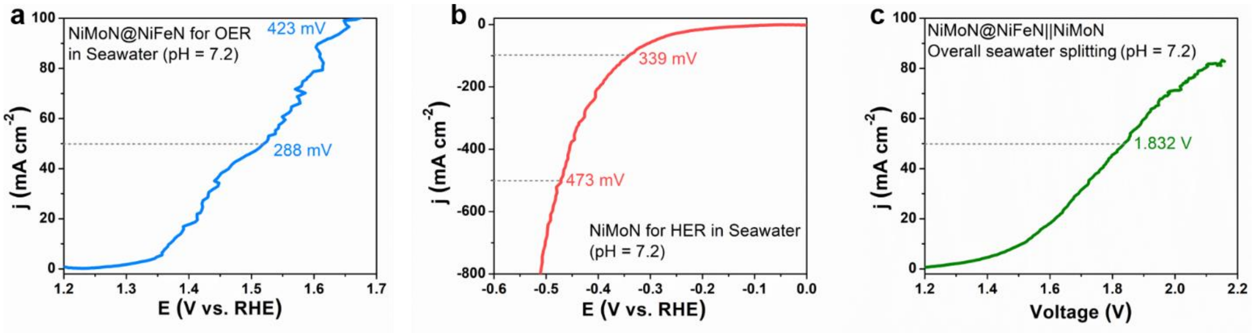

Supplementary Figure 20 | Overall seawater splitting performance in natural seawater. Polarization curves of (a) NiMoN@NiFeN for OER, (b) NiMoN for HER, and (c) NiMoN@NiFeN||NiMoN for overall seawater splitting using natural seawater as the electrolyte at 25 ℃.

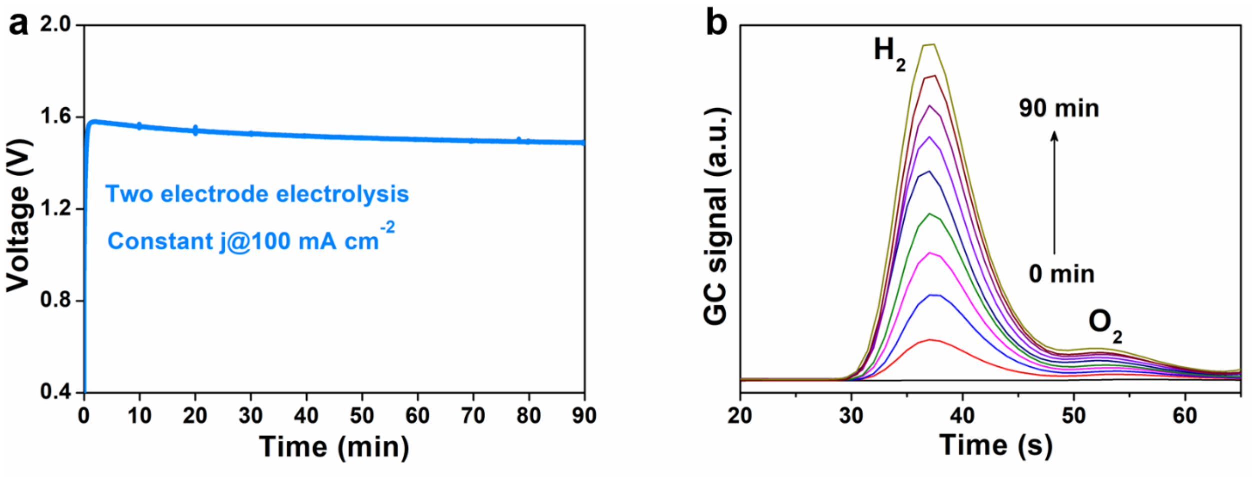

Supplementary Figure 21 | Measurements of H_2 and O_2 amounts for FE calculation. (a) Chronopotentiometric curve of the NiMoN@NiFeN||NiMoN electrolyzer for overall seawater splitting at a constant current density of 100 mA cm^{-2} for the gas chromatography (GC) tests. Electrolyte: 1 M KOH + 0.5 M NaCl; temperature: 25 °C. (b) GC signals of H_2 and O_2 during 90 min GC testing with detection every 10 min.

Faradaic efficiency (FE) describes the transfer efficiency of electric charge in an electrochemical reaction system, and it is the ratio between the utilized charge by the reactants and the amount of total charge of the external circuits.[5] We calculated the FE by comparing the experimentally quantified amounts of H_2 and O_2 to the theoretically calculated gas amounts, resulting in an estimated average FE value of 97.8%.

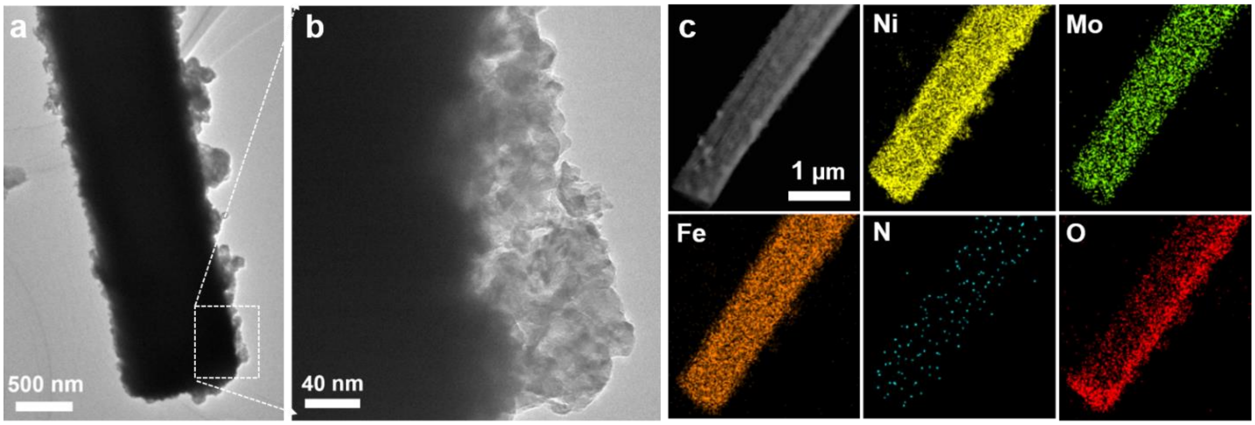

Supplementary Figure 22 | Nanostructure of NiMoN@NiFeN after seawater electrolysis. (a,b) TEM images at different magnifications, and (c) DF-STEM image and corresponding elemental mapping of NiMoN@NiFeN after 100 h seawater electrolysis at 500 mA cm^{-2} in 1 M KOH + Seawater.

To further study the stability of the 3D core-shell nanostructures during seawater electrolysis, we conducted TEM images of the NiMoN@NiFeN catalyst after 100 h seawater electrolysis at 500 mA cm^{-2} in 1 M KOH + Seawater. As shown in Supplementary Figs. 22a and 22b, many NiFeN nanoparticles remain closely attached on the NiMoN nanorod, demonstrating the integrity of the 3D core-shell nanostructure of NiMoN@NiFeN. The one small change is that the thickness of the NiFeN nanoparticles shows a slight decrease after 100 h seawater electrolysis. The DF-STEM and corresponding elemental mapping images in Supplementary Fig. 22c indicate the decreased N and increased O content on the NiMoN@NiFeN surface after seawater electrolysis, which is consistent with the results in Fig. 5d.

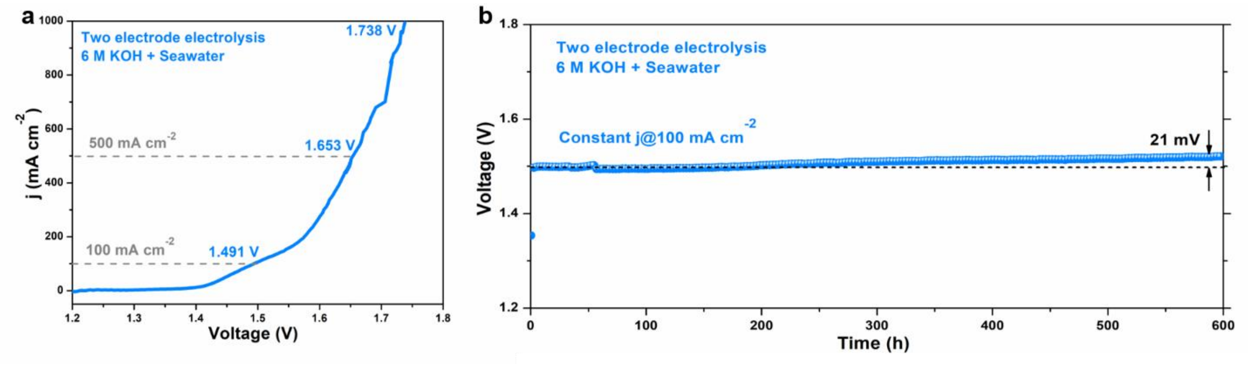

Supplementary Figure 23 | Overall seawater splitting performance in 6 M KOH + Seawater. (a) Polarization curve after iR compensation, and (b) durability test of the NiMoN@NiFeN||NiMoN electrolyzer at a constant current density of 100 mA cm^{-2} in 6 M KOH + Seawater (resistance: ~0.96 Ω) at 25 °C.

To further evaluate the performance of our catalysts toward commercial applications, we tested the activity of NiMoN@NiFeN||NiMoN for overall seawater splitting in a very harsh condition of 6 M KOH + Seawater at 25 °C, as well as its stability over 600 h in this condition. As the results shown in Supplementary Fig. 23a, our two-electrode cell achieves current densities of 100, 500, and 1000 mA cm^{-2} at very low voltages of 1.491, 1.653, and 1.738 V, respectively. More importantly, the voltage only increases about 21 mV after 600 h electrolysis at 100 mA cm^{-2} in 6 M KOH + Seawater (Supplementary Fig. 23b), further demonstrating the excellent stability. Therefore, the two catalysts are very active and robust for alkaline seawater splitting, exhibiting great potential for commercial applications.

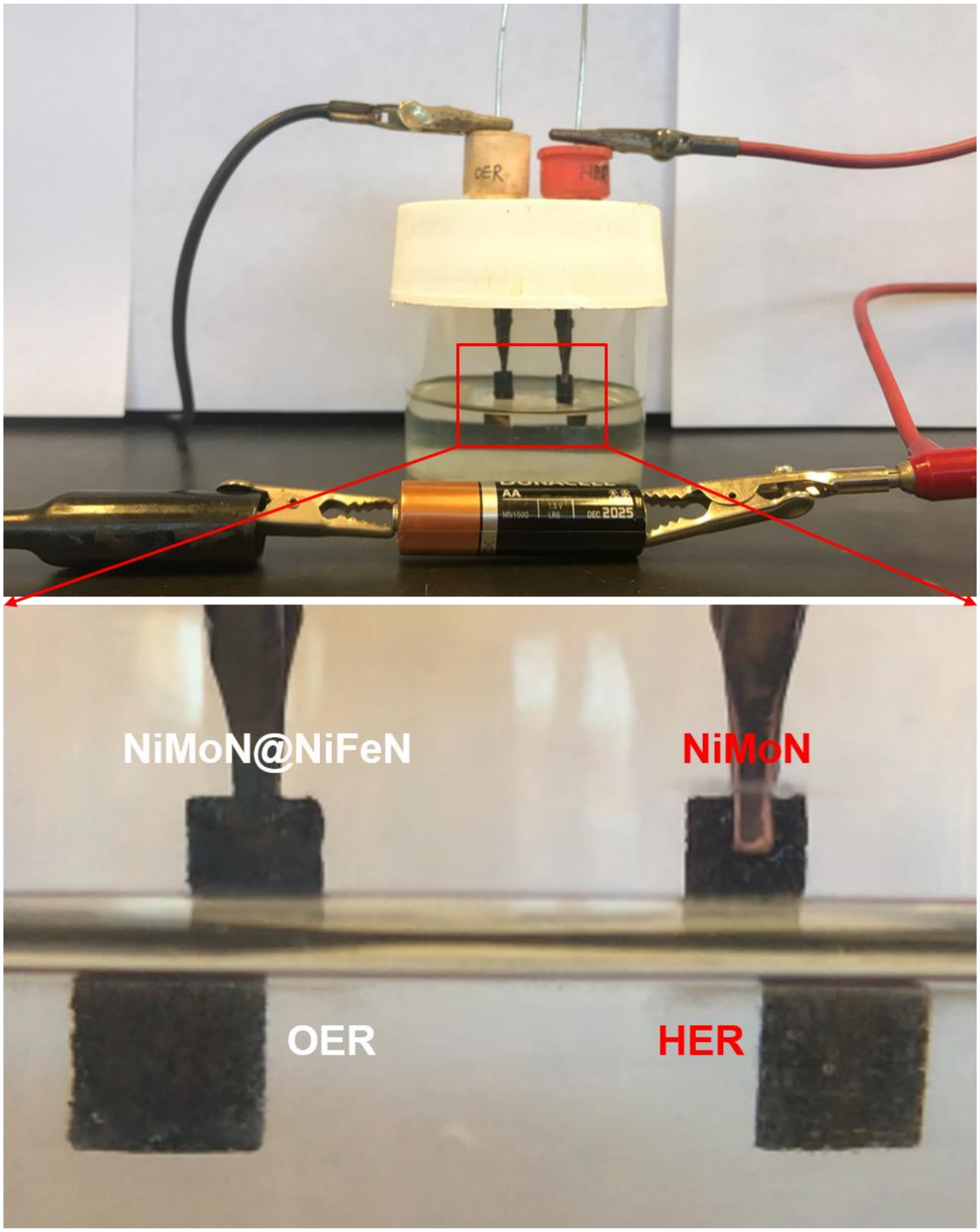

Supplementary Figure 24 | Photograph of gas bubbles. Photograph showing the O_2 and H_2 bubbles produced from overall seawater splitting driven by a 1.5 V AA battery. Electrolyte: 1 M KOH + 0.5 M NaCl; temperature: 25 °C.

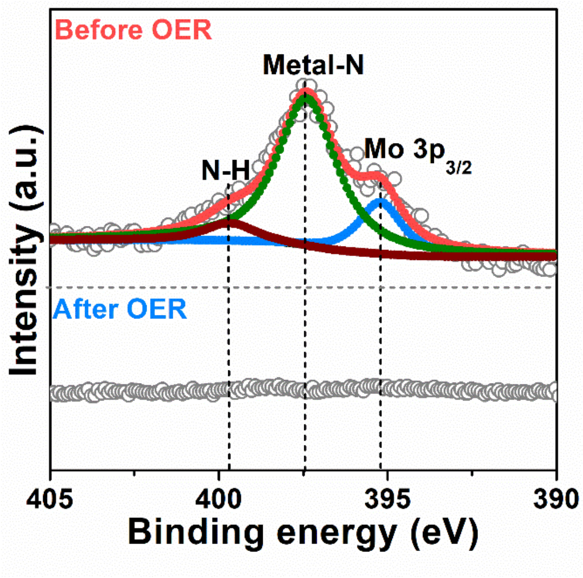

Supplementary Figure 25 | XPS of N 1s. High-resolution XPS of N 1s of NiMoN@NiFeN after OER test in comparison with that before OER test.

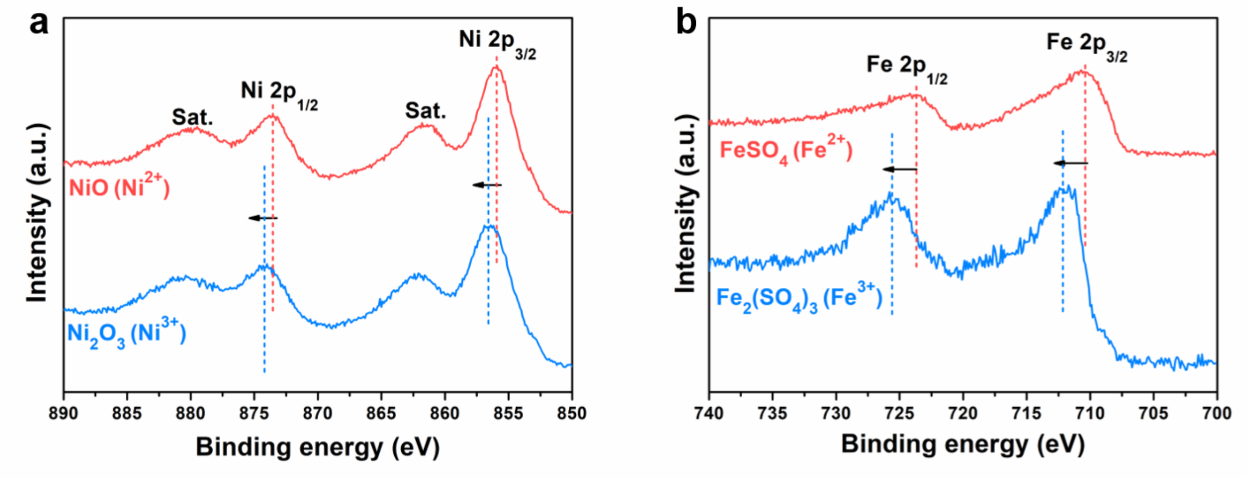

Supplementary Figure 26 | XPS spectrum. High-resolution XPS of (a) Ni 2p of NiO and Ni_2O_3, and (b) Fe 2p of $FeSO_4$ and $Fe_2(SO_4)_3$ for reference.

To study the oxidation states of Ni and Fe in the NiMoN@NiFeN catalyst, we measured the XPS of $FeSO_4$, $Fe_2(SO_4)_3$, NiO, and Ni_2O_3 for reference. The $FeSO_4$ and $Fe_2(SO_4)_3$ were commercial powders used as received and the NiO and Ni_2O_3 were synthesized from commercial Ni powders under different temperatures. As the results in Supplementary Fig. 26 show, the main peaks of $Fe_2(SO_4)_3$ (Fe^{3+}) and Ni_2O_3 (Ni^{3+}) positively shift toward higher binding energy compared with those of $FeSO_4$ (Fe^{2+}) and NiO (Ni^{2+}), which demonstrates that the higher valence states of Ni^{3+} and Fe^{3+} exhibit higher binding energy.

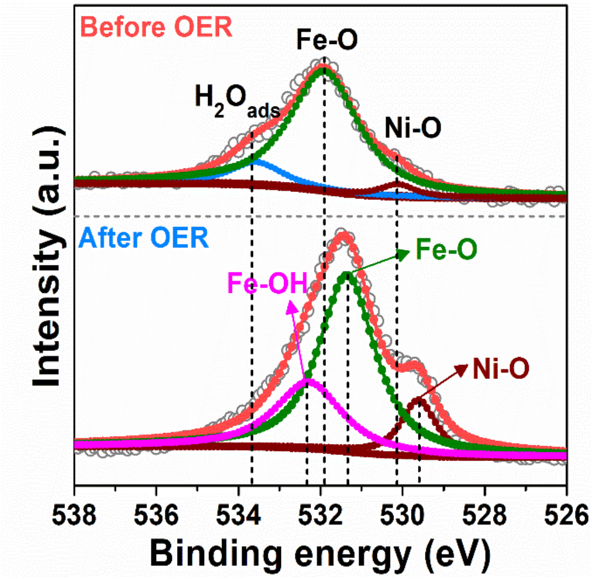

Supplementary Figure 27 | XPS of O 1s. High-resolution XPS of O 1s of NiMoN@NiFeN after OER test in comparison with that before OER test.

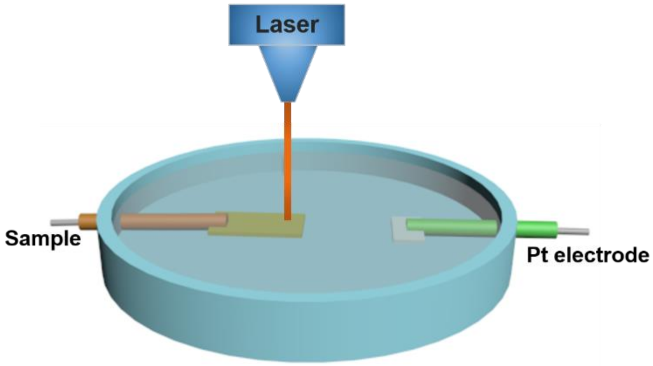

Supplementary Figure 28 | *In situ* Raman device. Schematic illustration of a home-made two-electrode cell for the *in situ* Raman measurement.

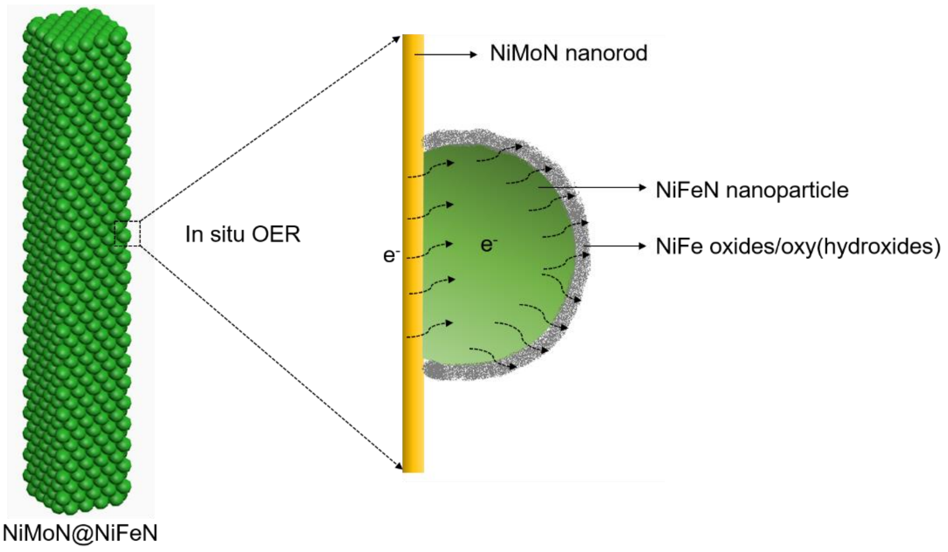

Supplementary Figure 29 | Multi-level core-shell nanostructure. Schematic illustration of the multi-level core-shell nanostructure of NiMoN@NiFeN during OER for efficient charge transfer.

Our NiMoN@NiFeN core-shell catalyst exhibits a multi-level core-shell nanostructure after OER. As the schematic illustration in Supplementary Fig. 29 shows, the NiMoN nanorod cores are robust and efficient supports for electron transfer to the NiFeN nanoparticles. Following transfer, the electrons then rapidly migrate to the surface active sites of the NiFe oxide/oxy(hydroxide) layers. In addition, such a multi-level core-shell nanostructure not only guarantees sufficient active sites for catalytic reactions, but also facilitates gaseous product release, which makes our catalyst OER active.

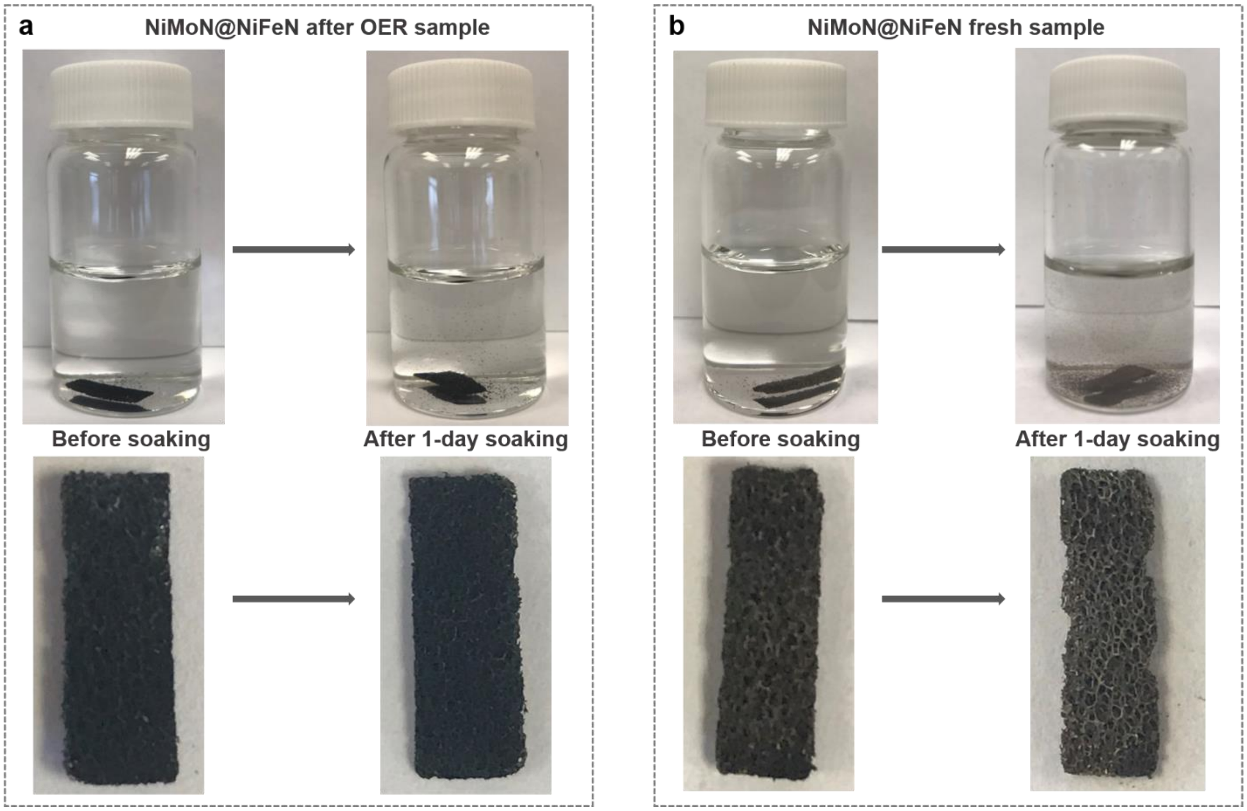

Supplementary Figure 30 | Corrosion resistance mechanism. Optical images of (a) a post-OER NiMoN@NiFeN sample, and (b) a fresh NiMoN@NiFeN sample before and after 1-day soaking in natural seawater.

The aggressive chloride anions in seawater can corrode many catalysts and substrates through metal chloride-hydroxide formation mechanisms:[6]

Adsorption of Cl^- by surface polarization: $M + Cl^- \rightarrow MCl_{ads} + e^-$

Dissolution by further coordination: $MCl_{ads} + Cl^- \rightarrow MCl_x^-$

Conversion from chloride to hydroxide: $MCl_x^- + OH^- \rightarrow M(OH)_x + Cl^-$

where M represents metal atom in the catalyst. Since there is metal chloride-hydroxide formation, the *in situ* evolved amorphous layers of NiFe oxide/oxy(hydroxide) that appear on the anode surface following OER may contribute to the good chlorine-corrosion resistance of the

NiMoN@NiFeN catalyst. To confirm this, we put two pieces of the NiMoN@NiFeN catalyst in seawater, one of which was a fresh sample before OER, and the other was a sample after OER, with NiFe oxy(hydroxide) formed on the surface. As the optical images in Supplementary Fig. 30 show, after 1-day soaking in seawater, the post-OER NiMoN@NiFeN sample shows very good structural integrity, appearing nearly the same as before soaking. In sharp contrast, the fresh NiMoN@NiFeN sample exhibits apparent corrosion, with many catalyst fragments appearing after 1-day soaking in seawater. Therefore, the *in situ* generated NiFe oxy(hydroxide) layers on the anode surface are critical to the stability against chloride corrosion.

Supplementary Tables

Supplementary Table 1 | OER activity comparison between the NiMoN@NiFeN catalyst and other reported non-noble metal electrocatalysts in 1 M KOH at room temperature. Here η_{100} and η_{500} correspond to the overpotentials required to achieve current densities of 100 and 500 mA cm^{-2}, respectively.

Catalyst	Support	η_{100} (mV)	η_{500} (mV)	TOF	Reference
NiMoN@NiFeN	Ni foam	277	337	0.09 s^{-1} at 300 mV	This work
$Zn_{0.2}Co_{0.8}OOH$	Glassy carbon	290*	NA	NA	7
Se-doped FeOOH	Fe foam	279	348	NA	8
NiCoFe-MOF	Ni foam	310*	NA	NA	9
FeNiP/NCH	Glassy carbon	340*	NA	0.25 s^{-1} at 300 mV	10
(Ni, Fe)OOH	Ni foam	220*	259	0.073 s^{-1} at 300 mV	11
$Fe_xCo_{1-x}OOH$	Ni foam	300*	NA	0.014 s^{-1} at 300 mV	12
NiFeRu LDH	Ni foam	260	NA	NA	13
FeP/Ni_2P	Ni foam	218*	270*	NA	14
NiFeV	Carbon paper	264	291	0.574 s^{-1} at 300 mV	15
$Co\text{-}Ni_3N$	Carbon cloth	385*	NA	0.013 s^{-1} at 350 mV	16
Cu@NiFe LDH	Cu foam	281	311	NA	17
NiFe LDH/graphene	Glassy carbon	325*	NA	NA	18
NiFe-OH/NiFeP	Ni foam	245	NA	0.036 s^{-1} at 250 mV	19
NiFeSe derived oxides	Ni foam	220*	283*	NA	20
FeCoW	Au foam	253*	NA	0.46 s^{-1} at 300 mV	21
NiFe LDH	Ni foam	450*	NA	NA	22

* Value calculated from the curve shown in the reference.

Supplementary Table 2 | OER activity comparison between the NiMoN@NiFeN catalyst and other reported non-noble metal electrocatalysts in different alkaline simulated and natural seawater, as well as neutral electrolytes at room temperature.

Catalyst	Electrolyte	j (mA cm^{-2})	η (mV)	Reference
NiMoN@NiFeN	1 M KOH + 0.5 NaCl	100	286	This work
		500	347	
		1000	377	
	1 M KOH + Seawater	100	307	
		500	369	
		1000	398	
NiFe/NiS$_x$-Ni	1 M KOH + 0.5 NaCl	400	~300	6
		1500	~380	
NiFe LDH	0.1 M KOH + 0.5 NaCl	10	359	23
	0.3 M Borate buffer + 0.5 NaCl	10	490	
Metal hexacyanometallates	1 M Phosphate buffer	48.5	~570	24
Fe-based film	0.1 M Phosphate buffer	5	~565	25
Co phosphate and borate	1 M Phosphate buffer	100	442	26
	1 M Borate buffer	100	363	

Supplementary References

(1) Zhuang, L. *et al.* Defect-induced Pt-Co-Se coordinated sites with highly asymmetrical electronic distribution for boosting oxygen-involving electrocatalysis. *Adv. Mater.* **31**, 1805581 (2019).

(2) Zou, X. *et al.* Ultrafast formation of amorphous bimetallic hydroxide films on 3D conductive sulfide nanoarrays for large-current-density oxygen evolution electrocatalysis. *Adv. Mater.* **29**, 1700404 (2017).

(3) Bates, M. K., Jia, Q., Doan, H., Liang, W. & Mukerjee, S. Charge-transfer effects in Ni-Fe and Ni-Fe-Co mixed-metal oxides for the alkaline oxygen evolution reaction. *ACS Catal.* **6**, 155-161 (2015).

(4) Jin, Y., Huang, S., Yue, X., Du, H. & Shen, P. K. Mo-and Fe-modified $Ni(OH)_2$/NiOOH nanosheets as highly active and stable electrocatalysts for oxygen evolution reaction. *ACS Catal.* **8**, 2359-2363 (2018).

(5) Wang, J., Xu, F., Jin, H., Chen, Y. & Wang, Y. Non-noble metal-based carbon composites in hydrogen evolution reaction: fundamentals to applications. *Adv. Mater.* **29**, 1605838 (2017).

(6) Kuang, Y. *et al.* Solar-driven, highly sustained splitting of seawater into hydrogen and oxygen fuels. *Proc. Natl Acad. Sci. USA* **116**, 6624-6629 (2019).

(7) Huang, Z.-F. *et al.* Chemical and structural origin of lattice oxygen oxidation in Co-Zn oxyhydroxide oxygen evolution electrocatalysts. *Nat. Energy* **4**, 329-338 (2019).

(8) Niu, S. *et al.* Se-doping activates FeOOH for cost-effective and efficient electrochemical water oxidation. *J. Am. Chem. Soc.* **141**, 7005-7013 (2019).

(9) Qian, Q., Li, Y., Liu, Y., Yu, L. & Zhang, G. Ambient fast synthesis and active sites deciphering of hierarchical foam-like trimetal-organic framework nanostructures as a platform for highly efficient oxygen evolution electrocatalysis. *Adv. Mater.* **31**, 1901139 (2019).

(10) Wei, Y.-S. *et al.* A single-crystal open-capsule metal-organic framework. *J. Am. Chem. Soc.* **141**, 7906-7916 (2019).

(11) Zhou, H. *et al.* Water splitting by electrolysis at high current densities under 1.6 volts. *Energy Environ. Sci.* **11**, 2858-2864 (2018).

(12) Ye, S. H., Shi, Z. X., Feng, J. X., Tong, Y. X. & Li, G. R. Activating CoOOH porous nanosheet arrays by partial iron substitution for efficient oxygen evolution reaction. *Angew. Chem. Int. Ed.* **57**, 2672-2676 (2018).

(13) Chen, G. *et al.* Accelerated hydrogen evolution kinetics on NiFe-layered double hydroxide electrocatalysts by tailoring water dissociation active sites. *Adv. Mater.* **30**, 1706279 (2018).

(14) Yu, F. *et al.* High-performance bifunctional porous non-noble metal phosphide catalyst for overall water splitting. *Nat. Commun.* **9**, 2551 (2018).

(15) Jiang, J. *et al.* Atomic-level insight into super-efficient electrocatalytic oxygen evolution on iron and vanadium co-doped nickel (oxy) hydroxide. *Nat. Commun.* **9**, 2885 (2018).

(16) Zhu, C. *et al.* In situ grown epitaxial heterojunction exhibits high-performance electrocatalytic water splitting. *Adv. Mater.* **30**, 1705516 (2018).

(17) Yu, L. *et al.* Cu nanowires shelled with NiFe layered double hydroxide nanosheets as bifunctional electrocatalysts for overall water splitting. *Energy Environ. Sci.* **10**, 1820-1827 (2017).

(18) Jia, Y. *et al.* A heterostructure coupling of exfoliated Ni-Fe hydroxide nanosheet and defective graphene as a bifunctional electrocatalyst for overall water splitting. *Adv. Mater.* **29**, 1700017 (2017).

(19) Liang, H. *et al.* Amorphous NiFe-OH/NiFeP electrocatalyst fabricated at low temperature for water oxidation applications. *ACS Energy Lett.* **2**, 1035-1042 (2017).

(20) Xu, X., Song, F. & Hu, X. A nickel iron diselenide-derived efficient oxygen-evolution catalyst. *Nat. Commun.* **7**, 12324 (2016).

(21) Zhang, B. *et al.* Homogeneously dispersed multimetal oxygen-evolving catalysts. *Science* **352**, 333-337 (2016).

(22) Luo, J. *et al.* Water photolysis at 12.3% efficiency via perovskite photovoltaics and earth-abundant catalysts. *Science* **345**, 1593-1596 (2014).

(23) Dionigi, F., Reier, T., Pawolek, Z., Gliech, M. & Strasser, P. Design criteria, operating conditions, and nickel-iron hydroxide catalyst materials for selective seawater electrolysis. *ChemSusChem* **9**, 962-972 (2016).

(24) Hsu, S. H. *et al.* An earth-abundant catalyst-based seawater photoelectrolysis system with 17.9% solar-to-hydrogen efficiency. *Adv. Mater.* **30**, 1707261 (2018).

(25) Wu, Y. *et al.* Fast and simple preparation of iron-based thin films as highly efficient water-oxidation catalysts in neutral aqueous solution. *Angew. Chem. Int. Ed.* **54**, 4870-4875 (2015).

(26) Esswein, A. J., Surendranath, Y., Reece, S. Y. & Nocera, D. G. Highly active cobalt phosphate and borate based oxygen evolving catalysts operating in neutral and natural waters. *Energy Environ. Sci.* **4**, 499-504 (2011).
